# Species Difference? Bovine, Trout, and Human Plasma Protein Binding of Per- and Polyfluoroalkyl Substances

**DOI:** 10.1021/acs.est.3c10824

**Published:** 2024-05-28

**Authors:** Weiping Qin, Beate I. Escher, Julia Huchthausen, Qiuguo Fu, Luise Henneberger

**Affiliations:** † Department of Cell Toxicology, UFZ—Helmholtz Centre for Environmental Research, 04318 Leipzig, Germany; ‡ Environmental Toxicology, Department of Geosciences, Eberhard Karls University Tübingen, Schnarrenbergstr. 94-96, DE-72076 Tübingen, Germany; § Department of Environmental Analytical Chemistry, UFZ—Helmholtz Centre for Environmental Research, 04318 Leipzig, Germany

**Keywords:** PFAS, solid-phase microextraction, plasma binding mechanism, proteins and lipids, specific and nonspecific protein binding

## Abstract

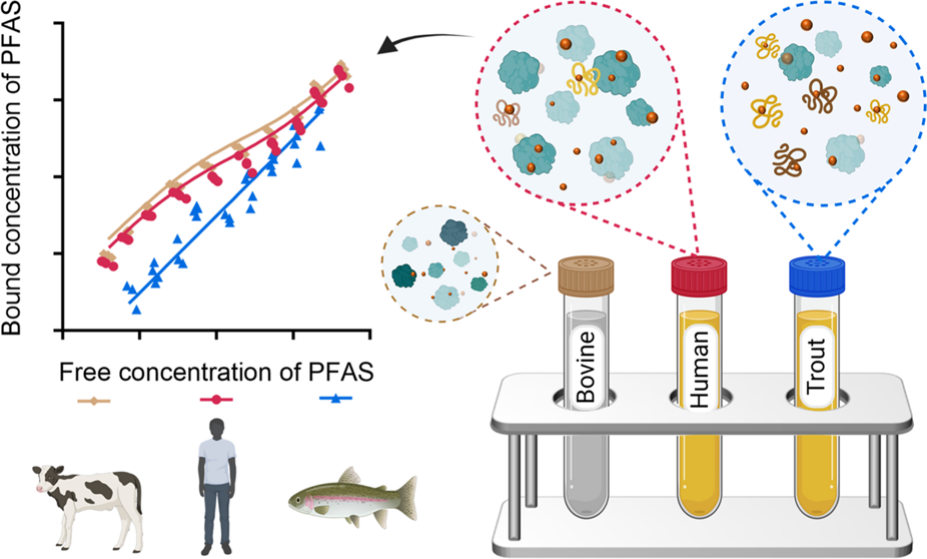

Per- and polyfluoroalkyl substances (PFAS) strongly bind to proteins and lipids in blood, which govern their accumulation and distribution in organisms. Understanding the plasma binding mechanism and species differences will facilitate the quantitative *in vitro*-to-*in vivo* extrapolation and improve risk assessment of PFAS. We studied the binding mechanism of 16 PFAS to bovine serum albumin (BSA), trout, and human plasma using solid-phase microextraction. Binding of anionic PFAS to BSA and human plasma was found to be highly concentration-dependent, while trout plasma binding was linear for the majority of the tested PFAS. At a molar ratio of PFAS to protein ν < 0.1 mol_PFAS_/mol_protein_, the specific protein binding of anionic PFAS dominated their human plasma binding. This would be the scenario for physiological conditions (ν < 0.01), whereas in *in vitro* assays, PFAS are often dosed in excess (ν > 1) and nonspecific binding becomes dominant. BSA was shown to serve as a good surrogate for human plasma. As trout plasma contains more lipids, the nonspecific binding to lipids affected the affinities of PFAS for trout plasma. Mass balance models that are parameterized with the protein–water and lipid–water partitioning constants (chemical characteristics), as well as the protein and lipid contents of the plasma (species characteristics), were successfully used to predict the binding to human and trout plasma.

## Abstract 2

Differences in the binding behavior of PFAS between human and trout plasma were explained by differences in lipid and protein contents.

## 1 Introduction

Blood is one of the major carriers for many per- and polyfluoroalkyl substances (PFAS) in human beings^1^ and animal species.^2^ The freely dissolved and protein-bound PFAS in blood can be transported with the blood flow to tissues and organs.^3,4^ Binding of PFAS to blood components is reversible. Competitive binding between human serum albumin and organ-specific proteins^5^ may result in the selective accumulation of PFAS in specific tissues and organs. PFAS accumulate in liver,^6^ and even more alarming are detections of PFAS in brain^7,8^ and umbilical cord blood,^9,10^ indicating that PFAS can cross the blood–brain and placental barrier due to their high cell membrane permeability.^11^ Unlike most persistent organic pollutants (POPs) that mainly accumulate in the lipid phase, PFAS have high affinities to both lipids and proteins.^12^ Therefore, understanding the binding of PFAS to blood components (e.g., lipids and proteins) is crucial for the prediction of the distribution of PFAS in the body and improving the health risk assessment of PFAS.

The unbound fraction in plasma is an important input parameter for the simulation of the absorption, distribution, metabolism, and excretion (ADME) of PFAS via physiologically based pharmacokinetic (PBPK) modeling.^6,13^ The ratio of the bound and free concentrations in plasma is defined by the partition constant between plasma and water (*K*_plasma/w_). There are now more than 14 000 PFAS chemicals in the CompTox Chemistry Dashboard with different structures and speciation.^14^ PFAS may be present as different molecular species at a physiological pH of approximately 7.4. Perfluoroalkyl carboxylic acids (PFCAs) and perfluoroalkyl sulfonic acids (PFSAs) are fully deprotonated and anionic at this pH. The distribution ratio *D*_plasma/w_ at pH = 7.4 should be used for ionizable PFAS. The unbound fraction of PFAS is available for redistribution or excretion, while the bound fraction of PFAS in tissues and organs has raised concerns about the bioaccumulation and chronic exposure.^13^

To facilitate the quantitative *in vitro*-to-*in vivo* extrapolation (QIVIVE) for PFAS, the concentration–response curves from *in vitro* bioassays should be derived with free concentrations of PFAS to obtain freely dissolved effect concentrations, which can be compared to the actual PFAS levels that human are exposed to (i.e., freely dissolved concentrations in human plasma).^15−18^ Fetal bovine serum (FBS) is typically used as the nutrient supply in an *in vitro* cell-based bioassay, while fish and mice are common *in vivo* animal models. To make the results from the different *in vitro* and *in vivo* models comparable and to allow extrapolation to humans, plasma binding of PFAS among different species needs to be known, but it has not been assessed for PFAS systematically so far.

Human biomonitoring studies suggested that the human blood concentrations of PFAS were at the nanomolar level,^19^ while toxicological studies detected the biological effects of PFAS at widely different concentration ranges from upper nano- to millimolar concentrations.^20^ For example, a mean value of 40 nmol/L perfluorooctanoic acid (PFOA) in the plasma of breastfed children was associated with reduced antibody responses to childhood vaccines, e.g., production of interferon γ by lymphocytes.^21^ A reduction of interferon was found to be regulated via nuclear factor kappa B pathways in zebrafish after a 21 day exposure of PFOA at 2 μmol/L.^22^ Other mechanisms of immunotoxicity were proved by *in vivo* animal models after week- or month-administration of PFAS at mg/kg levels, as well as *in vitro* cell models under acute stimulation by PFAS in the μmol/L concentration range.^23^ As binding of anionic PFAS to proteins in bioassay medium and human plasma is highly concentration-dependent,^15^ the typically large differences between exposure and effect concentrations will have an impact on QIVIVE.

Equilibrium dialysis is widely used to determine the binding of chemicals to BSA and human serum albumin.^24−28^ The binding of anionic PFAS to different types of albumin was also identified by ligand blotting,^29^ mass spectrometry,^28^ and spectroscopy.^24^ However, the binding or dissociation constants of PFAS to albumin were derived from single concentrations or limited concentration ranges, which limits an overall understanding of the nonlinear binding behavior of PFAS. Bischel et al.^28^ depicted nonlinear binding curves with PFOA and PFNA in a concentration range from 1.6 to 2700 μM, but they only provided specific binding constants at a physiological PFAS:protein molar ratio (ν < 0.001 mol_PFAS_/mol_protein_). Solid-phase microextraction (SPME) has been used to develop binding isotherms of ionizable chemicals with a small volume of samples over 4 orders of magnitude in concentrations,^30^ where the specific and nonspecific binding constants can be differentiated by modeling.^15^

In the present study, we studied the binding mechanism of 16 PFAS to BSA, which was used as a reference for analyzing binding behaviors of PFAS to trout and human plasma. The 16 PFAS covered a wide range of chemical classes including seven PFCAs, two PFSAs, one fluorotelomer sulfonic acid (FTSA), one sulfonamide, three fluorotelomer alcohols (FTOHs), and two fluorinated pesticides with the individual C*_n_*F_2*n*_ group. Protein and plasma binding isotherms of 13 nonvolatile PFAS were measured in a high-throughput format using a BioSPME 96-Pin Device combined with liquid chromatography mass spectrometry (LCMS).^31^ Protein and plasma partition constants of three semivolatile FTOHs were measured with headspace (HS)-SPME combined with gas chromatography mass spectrometry (GCMS). Mass balance models (MBMs) were developed to describe plasma binding from system parameters and chemical-specific parameters. System parameters were volume fractions of proteins and lipids in different plasmas that were experimentally quantified. Chemical-specific parameters were the measured binding constants to the surrogate protein BSA and lipid–water distribution ratios from the literature.

## 2 Materials and Methods

### 2.1 Materials

Sixteen PFAS (perfluorobutanoic acid (PFBA), perfluorohexanoic acid (PFHxA), perfluoroheptanoic acid (PFHpA), perfluorooctanoic acid (PFOA), perfluorononanoic acid (PFNA), perfluoroundecanoic acid (PFUnA), perfluoro-2-methyl-3-oxahexanoic acid (HFPO–DA), perfluorohexanesulfonic acid (PFHxS), perfluorooctanesulfonic acid (PFOS), 6:2 fluorotelomer sulfonic acid (6:2 FTSA), perfluorooctane sulfonamide (PFOSA), 2-perfluorohexyl-ethanol (6:2 FTOH), 2-perfluorooctyl-ethanol (8:2 FTOH), 2-perfluorodecyl-ethanol (10:2 FTOH), hexaflumuron and flubendiamide) were investigated (Table S1). All PFAS were dissolved in methanol (1428, Chemsolute) as a stock solution. Acetonitrile (34863, Honeywell) and formic acid (Honeywell) were used as eluents for sample measurements. Bovine serum albumin (BSA, 05470, Sigma-Aldrich), trout, and human plasma (S4189, Biowest) were used for protein and plasma binding assays. Rainbow trout (*Oncorhynchus mykiss*) plasma was kindly provided by Pavel Šauer from the University of South Bohemia in České Budějovice, Czech Republic.

Plastic (7696548, Labsolute) and glass-coated (60180-P336, Labsolute) 96-deep-well plates and Supelco BioSPME 96-Pin Devices (59683-U, Sigma-Aldrich) coated with C18 particles were used for 13 nonvolatile PFAS. Headspace crimp vials (20 mL, 762926, Labsolute), a reassembled cover with a magnetic cap (44512, Wicom), and aluminum-coated silicone septa (6086772, Labsolute) and PDMS/DVB fiber (57345-U, Sigma-Aldrich) were used in SPME assays for 3 semivolatile FTOHs. Experiments of uptake kinetics and sorption isotherms were carried out for method development and validations. Experiments of BSA and plasma binding were carried out to derive the binding isotherms and constants.

### 2.2 Uptake Kinetics of PFAS into C18 Coating of BioSPME

Five or 10 mg of each PFAS was dissolved in 1 mL of methanol as stock solutions. PFAS solution was prepared by diluting the methanolic stock solution with phosphate-buffered saline (PBS) to the concentrations listed in Table S2. The pH value of the PFAS solution was adjusted to 7.4 using sodium hydroxide for each acidic PFAS. The methanol content in the PFAS solution was always ≤1%. Three aliquots of 600 μL were filled in the first 96-deep well plate, and 600 μL of desorption solvents was filled in the second 96-deep well plate. Glass-coated 96-deep well plates were used for hydrophobic PFUnA, PFOSA, hexaflumuron, and flubendiamide to avoid loss due to binding to plastic, and plastic 96-deep well plates were used for the other 9 PFAS (Table S2). The total concentrations of PFAS samples were quantified by a 1260 Infinity liquid chromatograph coupled with a 6420 Triple Quad mass spectrometer (LCMS, Agilent, USA) for mass balance before SPME. The detailed LCMS parameters can be found in Table S3.

The experimental process of BioSPME conditioning, PFAS extraction from the aqueous solution, and desorption of the PFAS from BioSPME were performed automated by a Hamilton Star Robot (Bonaduz, Switzerland) as described by Huchthausen et al.^32^ Briefly, the BioSPME 96-Pin Device was conditioned in isopropanol for 20 min and then in Milli-Q water for 10 s. The extraction and desorption processes were performed on a high-speed shaker with a shaking speed of 1000 rpm. The temperature of the shaker was set to 37 °C for extraction and room temperature (25 °C) for the desorption. The shortest extraction time was 10 min, and the desorption time was always 20 min. After the first cycle, the extracted PFAS solution and desorption solutions were transferred to a third 96-deep well plate for instrumental analysis. The whole process was repeated for different extraction times (20, 40, 80, and 120 min). The experimental device is not airtight, and the extraction time should not be longer than 120 min to avoid the evaporation of the sample. After all of the samples were collected, the PFAS concentrations in the extracted aqueous solution and in the desorption solutions were measured by LCMS.

### 2.3 Sorption Isotherms for BioSPME

Methanolic stock solutions (10 or 5 mg/mL) containing the individual PFAS were diluted with methanol first to 100 times the desired concentration, and then 50 μL of this solution was further diluted with 4950 μL of PBS (Figure S1). The concentrations of each PFAS (Table S4) were designed according to the distribution ratio of PFAS between the pin coating and water (*D*_pin/w_). For each PFAS, a 9-step dilution series was prepared from the sample with the highest concentration with a factor of 2 difference between each step (Figure S1). Different volumes of the sample with the highest concentration were added to a new vial, and the respective volume of PBS was added to achieve a final volume of 2 mL for each sample. All samples were vortexed for 30 s. Two aliquots of 600 μL for each concentration were filled in the first 96-deep well plate, and 600 μL of desorption solvents was filled in the second 96-deep well plate. The experimental process for BioSPME was the same as above, and detailed experimental conditions for each PFAS can be found in Table S2 (e.g., extraction time, type of desorption solvent, material of the 96-deep well plate used for desorption and extraction).

### 2.4 BSA and Plasma Binding Isotherms of 13 Nonvolatile PFAS

BSA solution was prepared by dissolving BSA in PBS. The sample preparation was the same as shown in Figure S1 but using a BSA solution for dilution. The concentrations of PFAS and BSA (Table S5) were designed individually to have bound fractions of PFAS to BSA within a range of 30–90% based on experimental results in the pretests. All PFAS samples with BSA were incubated at 37 °C and shaken at 250 rpm overnight to allow for equilibration of protein binding. On the second day, samples were transferred to a 96-deep well plate for BioSPME.

For human and trout plasma binding assays, the appropriate plasma concentration was prepared by diluting the plasma with PBS. The volumes of human and trout plasma were chosen for each PFAS to keep a similar plasma protein level to that for the BSA binding assays (Table S5). The sample preparation was the same as that in Figure S1 with diluted plasma. PFAS samples with human plasma were incubated at 37 °C, the trout plasma samples were incubated at room temperature (25 °C), and all samples were shaken at 250 rpm overnight for equilibration of plasma binding before BioSPME.

### 2.5 BSA and Plasma Binding of 3 Semivolatile FTOHs

Ten mg of the individual FTOHs were dissolved in 1 mL of methanol as stock solution. FTOH stock solutions were further diluted with methanol to different concentrations (Figure S2), and then 50 μL of methanolic solution was added to 4950 μL of BSA in PBS in a 20 mL headspace crimp vial. A reassembled cover with a magnetic cap and aluminum-coated silicone septa was secured to the vial immediately using a crimper to form a sealed space to avoid the loss of FTOHs. Samples in the vial were vortexed for 30 s and then incubated at 37 °C and 250 rpm for 2 h. FTOHs were extracted from the headspace using a PDMS/DVB fiber, and the concentrations of FTOHs were quantified by an 8890 gas chromatograph coupled to a 5977B GC/MSD (Agilent, Waldbronn, Germany). Detailed parameters for HS-SPME combined with GCMS are found in Table S6.

For protein and plasma binding assays, 10 mg/mL of BSA was selected to ensure a 30–90% fraction bound for the tested FTOHs (Table S5) according to the pretests. 100 mL/L of plasma was used to keep similar plasma protein levels as for the BSA binding assays. The sample preparation was the same as that in Figure S2 but using PBS-diluted BSA or plasma. FTOH samples with BSA and human plasma were incubated at 37 °C and shaken at 250 rpm for 2 h for equilibration of BSA and plasma binding, while samples with trout plasma were incubated at room temperature (25 °C) and shaken at 250 rpm for 2 h before measurements by HS-SPME combined with GCMS.

### 2.6 Protein and Lipid Quantification

Plasma was diluted with PBS by a factor of 50 to ensure that the lipid and protein concentrations were within the calibration ranges. Pierce BCA Protein Assay Kit (23228, Thermo Scientific) was used to determine protein concentrations. The sulfophospho-vanillin reaction was used to determine the lipid concentrations as described previously.^33^ Units of protein and lipid were converted from mass concentration (mg/L) to volume concentration (mL/L) using a density of protein of 1.36 kg/L and a density of lipid of 1 kg/L.

### 2.7 Acidity Constant Determination

The p*K*_a_ of PFOSA at 25 °C and an ionic strength of 0.15 M KCl was determined with a cosolvent method with methanol according to Yasuda-Shedlovsky,^34,35^ and the p*K*_a_ values of flubendiamide and hexaflumuron were determined with the UV-metric method,^36^ using a Sirius T3 automated titrator (Pion) equipped with a glass Ag/AgCl pH electrode and a UV dip probe. A detailed description can be found in the literature.^37^

## 3 Data Evaluation

### 3.1 Mass Balance of BioSPME

The method development and validation of the BioSPME for PFAS were similar to C18-SPME using single fibers in our previous study.^15^ The amount of PFAS in the water phase (*n*_w_, eq 1) and the coating of the pins (*n*_pin_, eq 2) were obtained from the measured concentrations of PFAS in the extracted aqueous phase (*C*_w_) and in the desorption solvent (*C*_des_) and their corresponding volumes (*V*_w_ and *V*_des_). The volume of the C18 pin coating (*V*_pin_) was approximately 80 nL.^31^ The mass balance (eq 3) was calculated to validate the method
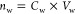
1
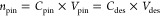
2
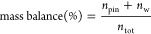
3

### 3.2 Uptake Kinetics into C18 Pin Coating of BioSPME

The equilibration times of PFAS between water and pin coating were determined from first-order kinetics (eqs 4 and 5), where *n*_w_ (eq 4) and *n*_pin_ (eq 5) are the amount of PFAS in the water phase and pin coating at different time points, respectively. *n*_w_ (*t*_0_) is the initial amount of PFAS used in the experiment. *k*_1_ is the rate constant for the decrease of the amount of the chemical in the water phase, and *k*_2_ is the apparent uptake rate constant to the pin coating^30^
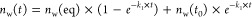
4
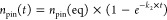
5

The time when sorption to the pin coating reached 95% equilibrium (*t*_0.95_) was calculated from *k*_2_ using eq 6
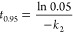
6

### 3.3 Freundlich-Type Model for Sorption Isotherms

Sorption isotherms of PFAS to the pin coating of the BioSPME, as well as to BSA and plasma proteins and lipids, were fitted with an empirical Freundlich adsorption isotherm by eq 7
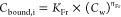
7

After a logarithmic transformation, the Freundlich-type model was derived with a linear relationship of the bound concentration, log *C*_bound,i_ (i = pin coating, BSA, or plasma protein and lipid), against the water concentration (log *C*_w_) by eq 8. The Freundlich constant log *K*_Fr_ and exponent *n*_Fr_ were adjusted by a best fit to the experimental data
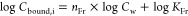
8

Distribution ratios between the sorption phases i and water, log *D*_i/w_ (eq 9), can be calculated at a given log *C*_bound,i_ (eq 10) or log *C*_w_ (eq 11) with log *K*_Fr_ and *n*_Fr_. The average value of log *D*_i/w_ is approximately equal to log *K*_Fr_ when the *n*_Fr_ is close to 1 canceling the log *C*_w_, suggesting that the log *D*_i/w_ is independent of concentrations. The standard error (SE) of log *K*_Fr_ (or log *D*_i/w_) was derived directly from the model fit. A 95% confidence interval (CI) was obtained as the values 1.96 × SE of either side of log *D*_i/w_
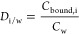
9
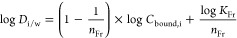
10
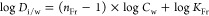
11

### 3.4 Mechanistic Model for BSA and Plasma Binding

The sorption isotherm of some anionic PFAS was concentration-dependent, which can be fitted nonlinearly with a combined binding/partitioning model.^15^ A wide range of molar ratios *ν* of bound PFAS-to-protein (eq 12, mol_PFAS_/mol_protein_) were used to identify the saturable binding range. A plateau of saturable binding in the range of *ν* < 1 by eq 13 suggests specific binding of PFAS with proteins
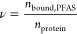
12
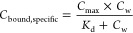
13

In the saturable binding, where there is only one binding site on the protein, the dissociation constant (*K*_d_) equals the equilibrium concentration of free PFAS (*C*_w_) required to occupy half of the maximum number of binding sites (*C*_max_) on the protein. The specific binding constant *D*_specific_ can be derived with the *C*_max_ and *K*_d_ by eq 14.^15^ The SE of *D*_specific_ was calculated by error propagation using the SE of *C*_max_ and *K*_d_ of the model fit. 95% CI was obtained as the values 1.96 × SE of either side of *D*_specific_
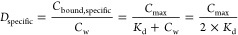
14

The nonspecific binding constant, *D*_nonspecific_, was derived by eq 15 with the fixed values of *C*_max_ and *K*_d_ from eq 13. The SE of *D*_nonspecific_ was derived from the model fitting. 95% CI was obtained as the values 1.96 × SE of either side of *D*_nonspecific_.
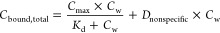
15

Protein and lipid in the plasma are the major sorption phases, with protein binding being highly specific at one binding site,^15^ while the nonspecific binding is relevant for proteins and lipids at higher concentrations. Therefore, the plasma binding isotherm was fitted by eq 16 that includes an extra term correcting for the ratio of the volume fraction of protein to protein plus lipid
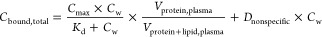
16

### 3.5 Mass Balance Model for Protein/Plasma Binding of FTOHs

In a closed headspace vial, the total amount of FTOH (*n*_tot_) partitions between water (*n*_w_), air (*n*_air_), wet-glass surface (*n*_glass_), and biomaterials (*n*_bound,i_, i = BSA, plasma proteins and lipids)
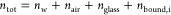
17

Partition constants of FTOHs between air and water (*K*_air/w_),^38^ wet-glass surface and air (*K*_glass/air_),^39^ and biomaterials and water (*D*_i/w_, i = BSA, plasma) were introduced into eq 17 to derive the concentration of FTOH in the aqueous phase of the PBS samples used as control, *C*_w_ (eq 18), as well as the aqueous phase of BSA and plasma samples, *C*_w,i_ (eq 19)
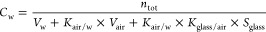
18
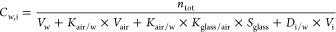
19

The distribution ratios, *D*_BSA/w_ and *D*_plasma/w_, can be derived from the peak areas of GCMS by using HS-SPME as described previously.^40^ Given a linear detector response (Figure S3), the GC peak areas of the FTOH from the control samples (*A*_w_, eq 20), BSA, or plasma samples (*A*_w,i_, eq 21) can be assumed to be linearly related to the concentration of the FTOH in the aqueous phases
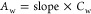
20
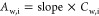
21

The slope is the response factor of the GC measurement, which cancels out if the ratio of peak areas (*A*_w_/*A*_w,i_) is calculated. Insertion of eqs 18–20 and eqs 19–21 also cancels out the *n*_tot_ for control samples and BSA and plasma samples. *D*_i/w_ was moved to the left side of the equation to yield eq 22. The SE of *D*_i/w_ was calculated from the standard deviation of samples measured with different concentrations. 95% CI was obtained as the values 1.96 × SE of either side of mean *D*_i/w_. The derivation of eq 22 and detailed information about the *K*_air/w_, *K*_glass/air_ (Table S7), and *S*_glass_ can be found in Supporting Information Text S1
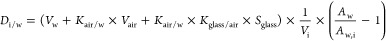
22

### 3.6 Plasma Binding Prediction

The distribution ratios of neutral PFAS (*D*_plasma/w_, pH = 7.4) between plasma proteins and lipids and water can be predicted by eq 23. *D*_BSA/w_ measured in the present study served as a proxy for protein distribution, as well as the distribution ratio of liposome and water *D*_lip/w_ for phospholipid distribution and olive oil and water *D*_oil/w_ as a proxy for neutral lipid distribution. The ratio of phospholipids to neutral lipids in human plasma is approximately 2:3 according to previous reports.^41,42^ The differentiation between phospholipids and neutral lipids is necessary because anionic chemicals showed high affinities to phospholipid^43^ but do not partition to neutral bulk lipids. Predictions of *D*_plasma/w_ for anionic PFAS can therefore be simplified by neglecting the third term in eq 23
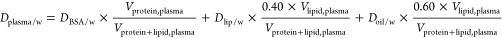
23

### 3.7 Statistical Analysis

Results were analyzed by Microsoft Excel and GraphPad Prism 10.0. The Freundlich-type model and combined binding/partitioning model were fitted with Graphpad Prism 10.0. The SE of the parameters derived from the model fitting is used to calculate the 95% confidence interval of the binding constants. Differences among testing concentrations were evaluated by Student’s *t* test. Results were considered as statistically significant if the *p* value was <0.05.

## 4 Results

### 4.1 Validation of BioSPME Method

The average mass balance of 13 PFAS measured by BioSPME was between 92 and 115% (eq 3, Table S2) in the kinetic uptake experiments, suggesting that the loss of chemicals to other compartments (e.g., plate or pin material) was less than 10%. As shown in Figure S4, 95% of equilibrium between pin coating and water (eq 6) was reached within 30 min for hydrophilic PFAS, while hydrophobic PFAS needed a longer time (max 58 min). Other experimental conditions, such as desorption solvents, desorption time, and plate materials, were determined for each PFAS according to their mass balance in the assays. Detailed information can be found in Table S2.

Sorption isotherms to the pin coating were fitted with a Freundlich-type model (eq 8). The isotherm curves of 8 anionic PFAS were found to be concentration-dependent, and thus, their log *D*_pin/w_ were fitted against log *C*_bound,pin_ (eq 10) and are listed in Table S4. The log *D*_pin/w_ were determined by setting *n*_Fr_ = 1 (eq 10) for long-chain PFNA, PFUnA, and PFOSA, as well as complex hexaflumuron and flubendiamide (Table S4), because their sorption isotherms were weakly dependent on concentrations (0.90 < *n*_Fr_ < 1) or independent of concentrations (*n*_Fr_ ≥ 1). Log *C*_pin_ of PFHpA was presented in a concentration-dependent way, although its *n*_Fr_ was 0.93, because the chain length of PFHpA is between PFHxA and PFOA, for which log *C*_pin_ was concentration-dependent.

### 4.2 BSA Binding Isotherms

**Figure 1:**
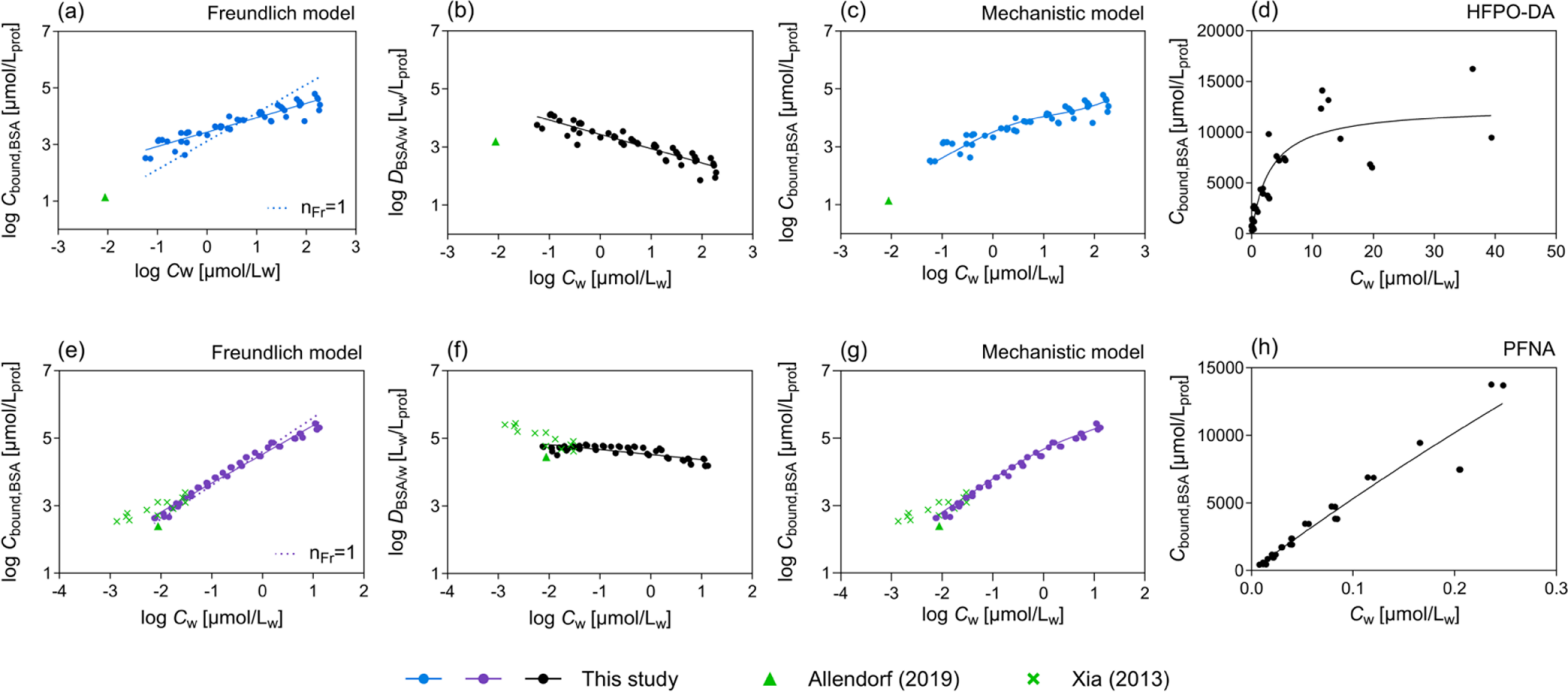
Bovine serum albumin (BSA) binding of (a–d) HFPO–DA and (e–h) PFNA. (a, e) Data points were fitted linearly with the Freundlich-type model (eq 8, solid line); the dotted line refers to fixed *n*_Fr_ = 1 for comparison. (b, f) The concentration-dependent distribution ratios between BSA and water, log *D*_BSA/w_, were fitted linearly (eq 11). (c, g) Experimental data points were fitted nonlinearly with the combined binding/partition model (eq 15). (d, h) The saturable specific binding in the low concentration range was derived with eq 13. Results of this study were compared with literature data^25,26^ (green triangle and crosses).

The BSA binding isotherms of the 13 PFAS were first fitted using the Freundlich-type model (eq 8, see Figure 1a,e for HFPO–DA and PFNA; for all other chemicals, see Figure S5). The log *D*_BSA/w_ were plotted against the log *C*_w_ (eq 11) and were concentration-dependent for HFPO–DA and PFNA (Figure 1b,f) and other 8 PFAS, and results of all PFAS are listed in Table 1. The BSA binding isotherm of PFUnA (0.90 < *n*_Fr_ < 1) was weakly dependent on concentrations, and the BSA binding isotherm of hexaflumuron and flubendiamide was independent of concentrations (*n*_Fr_ ≥ 1) (Table S8); therefore, their average log *D*_BSA/w_ were obtained by setting the *n*_Fr_ = 1 (eq 11).

**Table 1:** Distribution Ratios between BSA and Water (*D*_BSA/w_) and Distribution Ratios between Plasma and Water (*D*_plasma/w_) of 13 PFAS^a^

	BSA: log *D*_BSA/w_ [*L*_w_/*L*_prot_]	*R*^2^	human plasma: log *D*_plasma/w_ [*L*_w_/*L*_prot+lip_]	*R*^2^	trout plasma: log *D*_plasma/w_ [*L*_w_/*L*_prot+lip_]	*R*^2^
PFBA	log *D*_BSA/w_ = −0.298 log *C*_w_ + 2.91	0.60	log *D*_plasma/w_ = −0.402 log *C*_w_ + 2.80	0.68	1.43	0.74
PFHxA	log *D*_BSA/w_ = −0.271 log *C*_w_ + 3.43	0.88	log *D*_plasma/w_ = −0.287 log *C*_w_ + 3.29	0.84	2.49	0.91
PFHpA	log *D*_BSA/w_ = −0.305 log *C*_w_ + 3.95	0.81	log *D*_plasma/w_ = −0.389 log *C*_w_ + 3.79	0.89	3.32	0.93
PFOA	log *D*_BSA/w_ = −0.314 log *C*_w_ + 4.38	0.93	log *D*_plasma/w_ = −0.410 log *C*_w_ + 4.02	0.86	4.18	0.98
PFNA	log *D*_BSA/w_ = −0.147 log *C*_w_ + 4.52	0.61	log *D*_plasma/w_ = −0.183 log *C*_w_ + 4.13	0.91	4.71	0.98
PFUnA	4.75	0.96	4.54	0.95	4.99	0.98
HFPO–DA	log *D*_BSA/w_ = −0.493 log *C*_w_ + 3.44	0.86	log *D*_plasma/w_ = −0.633 log *C*_w_ + 3.41	0.95	log *D*_plasma/w_ = −0.646 log *C*_w_ + 3.20	0.97
PFHxS	log *D*_BSA/w_ = −0.472 log *C*_w_ + 4.28	0.92	log *D*_plasma/w_ = −0.677 log *C*_w_ + 3.95	0.96	log *D*_plasma/w_ = −0.236 log *C*_w_ + 3.96	0.68
PFOS	log *D*_BSA/w_ = −0.379 log *C*_w_ + 4.74	0.92	log *D*_plasma/w_ = −0.336 log *C*_w_ + 4.46	0.85	log *D*_plasma/w_ = −0.303 log *C*_w_ + 4.91	0.92
6:2 FTSA	log *D*_BSA/w_ = −0.092 log *C*_w_ + 3.86	0.54	log *D*_plasma/w_ = −0.144 log *C*_w_ + 3.66	0.71	4.11	0.91
PFOSA	log *D*_BSA/w_ = −0.105 log *C*_w_ + 4.28	0.50	log *D*_plasma/w_ = −0.103 log *C*_w_ + 3.87	0.52	log *D*_plasma/w_ = −0.164 log *C*_w_ + 4.26	0.60
hexaflumuron	3.96	0.97	4.29	0.94	4.61	0.91
flubendiamide	3.98	0.96	3.88	0.99	4.46	0.97

a Regression equations between log *D*_BSA/w_ and log *D*_plasma/w_ against log *C*_w_ were derived using a Freundlich-type model (eq 11). The concentration unit of PFAS in the water phase (*C*_w_) is micromolar [μmol/L].

For nonlinear binding isotherms (*n*_Fr_ < 0.9), a combined binding/partitioning model was used to derive the specific and nonspecific log *D*_BSA/w_ (Figure 1c,g). If a plateau of *C*_bound,BSA_ was found in the low concentration range (*ν* < 1, eq 13), e.g., for HFPO–DA (Figure 1d), specific binding applies in this concentration range and the specific *D*_BSA/w,specific_ (eq 14) was derived. The nonspecific log *D*_BSA/w,nonspecific_ was subsequently derived from the overall fit of the isotherm (eq 15). Similarly, the specific and nonspecific log *D*_BSA/w_ (Table 2) could be derived for PFBA, PFHxA, PFHpA, PFOA, PFHxS, and PFOS from the concentration-dependent binding isotherms (Figure S5).

However, for some PFAS such as PFNA (Figure 1h), 6:2 FTSA, and PFOSA (Figure S5h,i), no specific binding could be identified because there was no plateau of *C*_bound,BSA_ in saturable binding curves. An average log *D*_BSA/w_ was obtained for them by setting *n*_Fr_ = 1 (eq 11). This may be due to sensitivity limitations of the SPME method and instrumental analysis. All values of specific and nonspecific log *D*_BSA/w_ for 7 anionic PFAS, as well as average log *D*_BSA/w_ of other 6 PFAS, are listed in Table 2.

BSA binding isotherms of PFBA, PFOA, and PFHxS measured in the present study were compared with our previous results with C18-SPME using single fibers.^15^ Because the data from the two methods were almost overlapping (Figure S6a–c), all data were fitted together to derive *D*_BSA/w_ (Tables 2 and S9). The specific binding of PFOS measured with C18-SPME was a bit higher. However, 5 mg/mL of BSA was used for the C18-SPME, resulting in a bound fraction of PFOS > 99% in the low concentration range. The bound fraction reduced to 40–90% after adjusting BSA to 0.1 mg/mL in this study (Figure S6d and Table S10). log *D*_BSA/w_ of PFOS were also fitted with data from two methods, but several points in the low concentrations were excluded. Detailed information can be found in Supporting Information Text S2.

### 4.3 Acidity Constants

The BSA binding isotherm of PFOSA was slightly concentration-dependent (Figure S5i). We therefore measured its acidity constant. PFOSA was found to be an N-acid with a p*K*_a_ value of 8.77 ± 0.27, which means that 4.1% of PFOSA is anionic at pH 7.4. Sulfonamide pharmaceuticals typically have p*K*_a_ > 9, but the perfluorinated alkyl chain possibly stabilizes the anion and reduces the p*K*_a_ value. As binding of the anion is higher and usually specific, we can explain the observed nonlinearity of the sorption isotherm by the speciation of PFOSA. We also measured the p*K*_a_ of hexaflumuron and flubendiamide with the values of 9.11 ± 0.143 and 9.03 ± 0.10, which means these chemicals are 98% neutral and 2% anionic at pH = 7.4. The p*K*_a_ values of anionic PFAS and neutral FTOHs were not measured since they are 100% anionic or neutral at physiological pH = 7.4.

**Table 2:** Distribution Ratios of PFAS between Lipids, Proteins, Plasma, and Water^a^,^b^

				BSA: log *D*_BSA/w_ [*L*_w_/*L*_prot_]		human plasma: log *D*_plasma/w_ [*L*_w_/*L*_prot+lip_]		trout plasma: log *D*_plasma/w_ [*L*_w_/*L*_prot+lip_]	
	number of C–F	log *D*_lip/w_ [*L*_w_/*L*_lip_]	log *D*_oil/w_ [*L*_w_/*L*_oil_]	specific	nonspecific or average*	specific	nonspecific or average*	specific	nonspecific or average*
PFBA	3	1.00^c^	n/a	*2.44 (2.10–2.63)*	1.97 (1.90–2.04)	2.20 (0.87–2.49)	1.35 (1.23–1.44)	n/a	1.43 (1.28–1.58)*
PFHxA	5	2.32^d^	n/a	3.26 (2.98–3.42)	2.69 (2.66–2.72)	3.19 (3.01–3.31)	2.61 (2.56–2.66)	n/a	2.49 (2.41–2.57)*
PFHpA	6	2.91^d^	n/a	4.12 (3.87–4.27)	3.47 (3.42–3.52)	4.10 (3.98–4.20)	3.20 (3.15–3.24)	n/a	3.32 (3.25–3.39)*
PFOA	7	3.52^d^	n/a	4.58 (4.47–4.67)	3.88 (3.86–3.90)	4.45 (4.34–4.54)	3.65 (3.59–3.70)	n/a	4.18 (4.14–4.21)*
PFNA	8	4.25^d^	n/a	n/a	4.61 (4.56–4.65)*	n/a	4.19 (4.14–4.24)*	n/a	4.71 (4.65–4.76)*
PFUnA	10	4.54^d^	n/a	n/a	4.75 (4.71–4.80)*	n/a	4.54 (4.49–4.59)*	n/a	4.99 (4.96–5.03)*
HFPO–DA	5	2.41^d^	n/a	3.31 (2.95–3.50)	2.17 (2.05–2.26)	3.35 (2.77–3.58)	1.99 (1.93–2.04)	3.56 (3.08–3.78)	1.89 (1.67–2.04)
PFHxS	6	4.13^d^	n/a	5.02 (4.90–5.11)	3.58 (3.50–3.64)	4.98 (4.80–5.11)	2.92 (2.78–3.03)	n/a	4.00 (3.91–4.10)*
PFOS	8	4.89^d^	n/a	5.27 (4.84–5.48)	4.17 (4.14–4.20)	4.82 (4.41–5.03)	4.07 (4.04–4.11)	5.49 (4.88–5.74)	4.57 (4.54–4.60)
6:2 FTSA	6	3.87^e^	n/a	n/a	3.87 (3.84–3.90)*	n/a	3.67 (3.63–3.70)*	n/a	4.11 (4.04–4.19)*
PFOSA	8	4.94^e^	4.61^h^	n/a	4.32 (4.28–4.37)*	n/a	3.90 (3.86–3.93)*	n/a	4.33 (4.27–4.40)*
hexaflumuron	2	4.58^f^	5.19^h^	n/a	3.96 (3.93–4.00)*	n/a	4.29 (4.24–4.35)*	n/a	4.61 (4.53–4.69)*
flubendiamide	3	3.28^f^	4.91^h^	n/a	3.98 (3.93–4.02)*	n/a	3.88 (3.85–3.91)*	n/a	4.46 (4.42–4.50)*
6:2 FTOH	6	4.38^g^	3.81^g^	n/a	2.67 (2.54–2.77)*	n/a	2.73 (2.49–2.89)*	n/a	2.77 (2.49–2.94)*
8:2 FTOH	8	5.85^g^	4.55^g^	n/a	4.61 (4.48–4.72)*	n/a	4.55 (4.49–4.61)*	n/a	5.01 (4.90–5.10)*
10:2 FTOH	10	7.73^e^	6.03^e^	n/a	6.72 (6.44–6.89)*	n/a	6.21 (6.11–6.28)*	n/a	6.86 (6.68–6.99)*

a n/a: not available.

b Distribution ratios between membrane lipids (liposomes) or neutral lipids and water (log *D*_lip/w_ or log *D*_oil/w_) were from the literature or predicted. Distribution ratios between bovine serum albumin and water (log *D*_BSA/w_) and between human or trout plasma and water (log *D*_plasma/w_) were measured in this study. Average log *D*_i/w_ were calculated with the Freundlich-type model (eq 11). log *D*_i/w_ of specific and nonspecific binding were derived with the combined binding/partitioning model (eqs 13, 15, 16). In parentheses are the 95% confidence intervals.

c log *D*_lip/w_ were from Droge et al.^44^

d log *D*_lip/w_ were from Ebert et al.^11^

e log *D*_lip/w_ of 6:2 FTSA and PFOSA were predicted from the linear relationship of experimental log *D*_lip/w_ against the number of fluorinated carbons (Figure S10) by eq S12. log *D*_lip/w_ of 10:2 FTOH were predicted by eq S13. log *D*_oil/w_ of 10:2 FTOH were predicted by eq S14.

f log *D*_lip/w_ were predicted with COSMOtherm.^45^

g log *D*_lip/w_ and log *D*_oil/w_ (olive oil) were from Endo et al.^46^

h log *D*_oil/w_ approximated by the ionization-corrected octanol–water partition constant log *D* (pH 7.4) (ACD/percepta 14.51.0).

### 4.4 Protein and Lipid Contents of Human and Trout Plasma

The human plasma contained 42.25 mL/L of protein, almost 10 times higher than the lipid content of 4.46 mL/L. The trout plasma had a lower protein content of 15.46 mL/L and more lipids of 7.08 mL/L compared with the human plasma (Table 3).

**Table 3:** Volume Fractions (Vf) of Proteins and Lipids in Human and Fish Plasma

	Vf_protein_ [mL/L]	Vf_lipid_ [mL/L]
human plasma	42.25	4.46
fish plasma	15.46	7.08

### 4.5 Plasma Binding Isotherms

Similar to BSA binding, the human plasma binding of 10 PFAS was concentration-dependent (Table 1), but specific and nonspecific log *D*_plasma/w_ could be distinguished for only 7 anionic PFAS (Table 2). Average values of log *D*_plasma/w_ for PFNA, PFUnA, 6:2 FTSA, PFOSA, hexaflumuron, and flubendiamide were derived with fixed *n*_Fr_ = 1 (Table 2). The volume fraction of proteins was 10 times higher than that of lipids; therefore, proteins are expected to dominate the human plasma binding. Differently, HFPO–DA, PFHxS, PFOS, 6:2 FTSA, and PFOSA (*n*_Fr_ < 0.90, Table S8) were found to have concentration-dependent binding isotherms for trout plasma, but only for HFPO–DA and PFOS, the specific binding could be fitted (Table 2).

**Figure 2:**
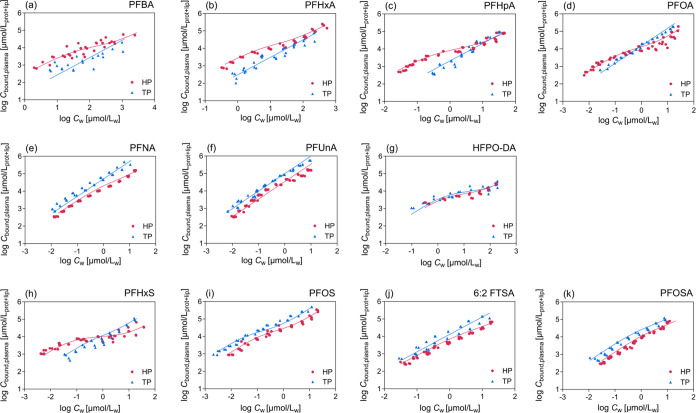
Human plasma (HP) and trout plasma (TP) binding isotherms of 11 anionic PFAS. Curves were fitted linearly with the Freundlich-type model (eq 8) or nonlinearly with the combined binding/partitioning model (eq 16). The selection of models was based on whether the binding isotherms were concentration-dependent (Table 1).

Human and trout plasma binding isotherms were directly compared for 11 of the 13 tested PFAS in Figure 2. Differences were observed between trout and human plasma binding in the low concentration ranges of PFBA, PFHxA, PFHpA, PFOA, and PFHxS, while the isotherms overlapped at high concentrations (Figure 2a–d,h) because the nonspecific lipid binding (log *D*_lip/w_) is similar to the nonspecific protein binding (log *D*_BSA/w_) (Table 2). The difference at low concentrations is due to the higher protein content of human plasma which led to dominance of strong specific binding. The trout plasma had lower protein content and higher lipid content and lipid binding may have masked the specific protein binding. For PFHxS and PFOA, trout plasma binding gradually surpassed the human plasma binding at high concentrations (Figure 2d,h), suggesting that lipid binding may dominate the plasma binding at high concentrations where their log *D*_lip/w_ were higher than the nonspecific log *D*_BSA/w_. For PFNA and 6:2 FTSA, trout plasma binding was linear, but slightly concentration-dependent for human plasma, indicating that specific protein binding was relevant but partially masked by nonspecific binding (Figure 2e,j). PFOSA showed a rather weak concentration dependence for both types of plasma (Figure 2k). Both plasma binding isotherms were linear for PFUnA (Figure 2f), hexaflumuron, and flubendiamide (Figure S7).

### 4.6 Comparison of BSA and Plasma Binding of Neutral FTOHs and Anionic PFAS

BSA binding of neutral 6:2 FTOH, 8:2 FTOH, and 10:2 FTOH was measured at four concentrations, and there was no significant difference (*t* test, *p* < 0.05) of log *D*_BSA/w_ among concentrations (Figure S8). Therefore, their log *D*_BSA/w_ values were calculated from the average values measured at different concentrations (Table 2). Similarly, the average values of log *D*_plasma/w_ of human and trout plasma were calculated for those chemicals that did not show any specific binding (Table 2). The log *D*_plasma/w_ of the FTOHs for trout plasma were higher than that of human plasma because FTOHs bind stronger to lipids compared to proteins (Table 2) and the volume fraction of lipids was higher in trout plasma than in human plasma. Both human and fish plasma binding constants of neutral FTOHs and anionic PFAS were chain-length-dependent (Figure S9).

The acidic functional groups have an impact on the specific binding of PFAS to BSA. Carboxylic and sulfonic acids deprotonate to anionic carboxylates and sulfonates and bind to proteins via electrostatic interaction, which may lead to the specific protein binding of PFBA, PFHxA, PFHpA, PFOA, HFPO–DA, PFHxS, and PFOS (Table 2). The BSA binding of 6:2 FTOH was 10 times lower than that of PFOA, PFHxS, and 6:2 FTSA, which have the same number of perfluorinated carbons, because the neutral alcohols bind to proteins mainly via van der Waals forces with contribution of hydrogen bonds by the alcohol groups. However, as the number of C–F increases, the hydrophobicity increases and consequently the nonspecific portion of binding dominated, where the specific binding of PFNA and PFUnA cannot be distinguished from the nonspecific binding (Figures 1h and S5e). Also, the impact of hydrophobicity may surpass that of the functional groups. For example, the log *D*_BSA/w_ (nonspecific or average) of PFNA, PFOS, PFOSA, and 8:2 FTOH with eight perfluorinated carbons were similar despite the different head groups. The log *D*_BSA/w_ of 10:2 FTOH were higher than that of PFUnA, both of which carry 10 perfluorinated carbons, presumably due to the combined effect of the extra ethane moiety (C_2_H_4_) of 10:2 FTOH and the different head groups of an anionic carboxylate versus a neutral hydroxy group (Table 2).

**Figure 3:**
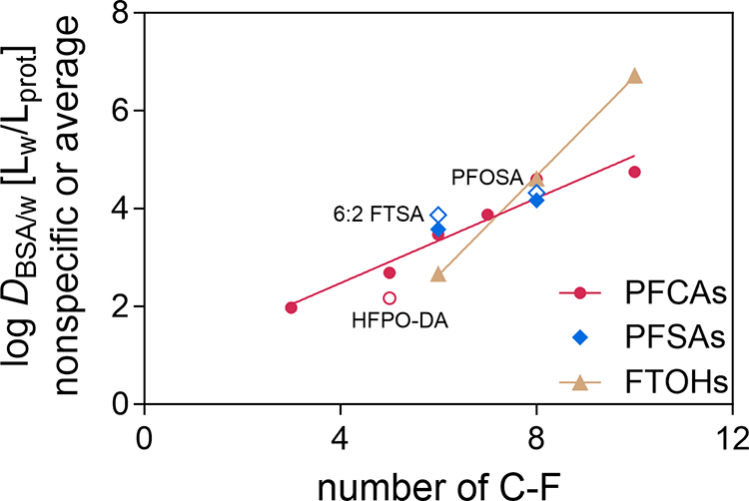
Nonspecific or average BSA binding, log *D*_BSA/w_ (pH = 7.4) of perfluoroalkyl carboxylic acids (PFCAs, magenta circle), sulfonic acids (PFSAs, blue diamond), and average BSA binding of fluorotelomer alcohols (FTOHs, gold triangle). log *D*_BSA/w_ of HFPO–DA (empty circle), 6:2 FTSA, and PFOSA (empty diamond) were excluded from the regression but plotted for comparison.

Linear regressions were developed for log *D*_BSA/w_ (nonspecific or average) against the number (*n*) of C–F for PFCAs and FTOHs (Figure 3) to study how the chain length may affect their binding constants (eqs 24 and 25). The relationship for PFSAs is missing because two values of log *D*_BSA/w_ of PFHxS and PFOS are not enough to fit an exclusive line for PFSAs. However, as shown in Figure 3, values of log *D*_BSA/w_ of PFHxS and PFOS overlapped with the regression of PFCAs. At high concentrations, hydrophobicity dominates the BSA binding of PFAS and the number of C–F has a more significant impact on the BSA binding than the functional groups of carboxylic and sulfonic acids. log *D*_BSA/w_ (nonspecific) of HFPO–DA, 6:2 FTSA, and PFOSA are excluded from the regression since their structures are different from PFCAs and PFSAs
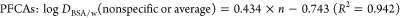
24
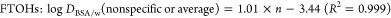
25

### 4.7 Prediction of Plasma Binding

Plasma binding of PFAS can be predicted by eq 23 by assuming that proteins and lipids in the plasma are the major sorption phases. Input parameters for the model are chemical properties (*D*_BSA/w_, *D*_lip/w_, and log *D*_oil/w_, Table 2), as well as volume fractions of proteins and lipids in different types of plasmas (Table 3).

Experimental values of *D*_lip/w_ and *D*_oil/w_ of PFCAs, PFSAs, and FTOHs were from the literature^11,44,46^ and are used to develop regression relationships of log *D*_lip/w_ or log *D*_oil/w_ against the number of C–F (Figure S10), which were further used to predict the log *D*_lip/w_ for 6:2 FTSA, FTOSA (eq S12), as well as log *D*_lip/w_ (eq S13) and log *D*_oil/w_ (eq S14) for 10:2 FTOH. The log *D*_lip/w_ of hexaflumuron and flubendiamide were predicted by COSMOtherm 2020^45^ because of their very different structures. For the partially charged PFOSA, flubendamide, and hexaflumuron (>95% neutral), we used the ionization-corrected octanol–water partition constant predicted with ACD as a proxy of log *D*_oil/w_. For the fully anionic PFAS, the partitioning to a neutral lipid was neglected.

**Figure 4:**
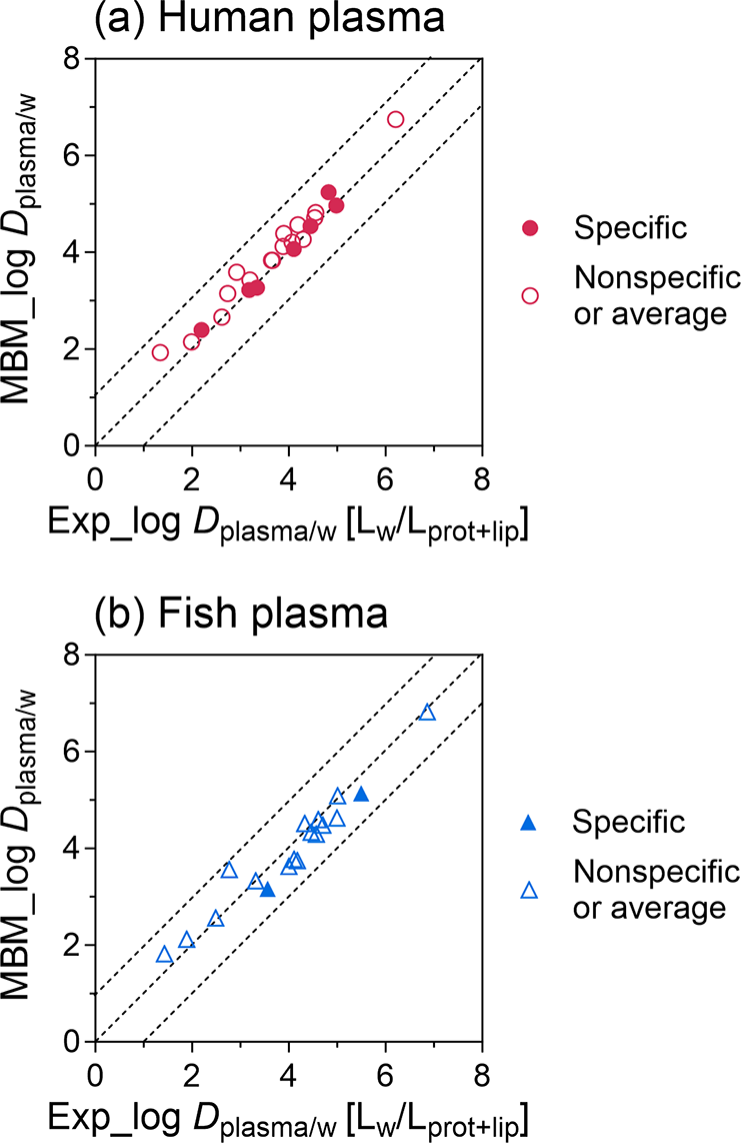
Prediction of plasma–water distribution ratios, log *D*_plasma/w_ (pH = 7.4), of 16 PFAS. log *D*_plasma/w_ of (a) human plasma or (b) trout plasma were measured experimentally (Exp) and compared with the *D*_plasma/w_ predicted by a mass balance model (MBM) from protein binding constants, log *D*_BAS/w_ (pH = 7.4) and lipid binding constants, log *D*_lip/w_ and log *D*_oil/w_, as well as the volume fractions of proteins and lipids in plasmas (eq 23).

The specific log *D*_plasma/w_ at low concentrations were predicted with log *D*_BSA/w_ (specific), and the nonspecific log *D*_plasma/w_ at high concentrations were predicted with log *D*_BSA/w_ (nonspecific or average). As shown in Figure 4, all of the predicted results were within a factor of 10 compared to the experimental ones for human and trout plasmas.

## 5 Discussion

### 5.1 Methods for Measuring BSA and Plasma Binding of PFAS

Serum albumin binding of PFAS has been measured by various methods in the past decades.^47^ Specific binding of PFAS to defined binding sites on certain proteins was identified in competition assays by using site-specific probes^48^ and probe-labeled proteins.^49^ Here, we compared binding constants of 9 PFAS measured by traditional dialysis,^25,26^ with the BSA binding isotherms measured in the present study (Figures 1 and S5). Literature data, which initially looked inconsistent, turned out to be located in different regions of the binding isotherms, reconciling results from different methods. The extensive binding isotherms derived in the present study depict a broader view of the binding behavior of these anionic PFAS.

The bound fraction affected the binding constants in this and previous studies.^24,25,27^ However, under actual physiological concentration, the molar ratio of PFAS to protein is low, suggesting that more than 99% PFAS would be bound in 100% plasma.^28^ It cannot be ruled out that the binding constants derived under the *in vitro* experimental conditions in the present study with a low plasma content may underestimate the bound fraction of some chemicals with very high affinities to proteins in the bloodstream *in vivo*. However, extrapolation from, e.g., 10% plasma should still be more accurate than measuring free concentrations at close to 100% bound fraction, which would be technically challenging to impossible.

Blood is a favorable matrix for an internal exposure assessment. Although plasma and serum are major fractions of the whole blood, the different components (e.g., blood cells, fibrinogen, platelet, and others) may affect the detected frequencies, concentrations, or distributions of PFAS.^50^ High-purity serum albumin is used in most mechanistic binding studies, while we compared the binding isotherms of BSA and plasma in the present study in order to further demonstrate the binding behavior of PFAS in real life. Plasma contains most of the proteins and also other components of blood after the removal of cells and clotting factors. Although proteins dominate the specific binding of plasma, the role of nonspecific binding to lipids cannot be ignored, especially for plasma with high lipid fraction like trout plasma.

### 5.2 Implications of Plasma Binding of PFAS for Organ-Specific Accumulation

Plasma binding of PFAS is chain-length-dependent (Figure S9), and log *D*_plasma/w_ > 4 were determined for PFOA, PFNA, PFUnA, PFHxS, and PFOS, indicating that they may accumulate in plasma and be transported in a bound form through the whole body. This can explain why middle- and long-chain PFAS were widely found in tissues and organs of humans,^3^ trouts,^4^ whales,^43^ and finless porpoises.^51^

The binding of PFAS to plasma components is reversible, and the free PFAS in plasma may redistribute to tissues and organ-specific proteins.^11^ The liver and brain have a higher metabolic demand and thus receive substantial blood flows. A competitive binding between human serum albumin and liver fatty acid-binding protein (hL-FABP) was found to correlate with the ratio of blood to liver concentration of PFAS.^52^ Differences in lipid homeostasis perturbation between mice and humans may also be partially related to (dose-dependent) differences in binding affinity.^52^

PFAS also have high affinities to transthyretin,^53,54^ which is primarily produced in the liver and also expressed in the choroid plexus of the brain.^55^ Competitive binding of PFAS between plasma components and transthyretin might also lead to the selective accumulation of PFAS in the liver and the brain.

Protein binding does not only affect internal distribution but also affect toxicokinetics, in particular, the elimination kinetics and mechanism. Their persistence, together with the high affinity to proteins in general and specifically liver fatty acid-binding proteins in the liver, can lead to slower clearance and consequently long half-lives (>1 year) of PFAS.^56−58^ Human urinary excretion was found to decrease with the chain length of PFCAs because only freely dissolved PFAS may be excreted via urine.^59^ Long-chain PFCAs are strongly bound and can only be eliminated via the bile to feces.^59^ With enterohepatic circulation and recycling of bile acids, PFCAs can also be reabsorbed back,^52,59,60^ slowing the elimination rate. Furthermore, it needs to be considered that half-lives of PFAS also depend on the activity of renal transporters and therefore knowledge of plasma protein binding alone is not sufficient to correctly predict PFAS half-lives.^61^

Although the values of *D*_plasma/w_ of FTOHs are noteworthy, especially 8:2 and 10:2 FTOH, their concentrations in human samples were very low or not detected^61^ because FTOHs can be metabolized to PFCAs (e.g., PFHxA, PFHpA, PFOA, PFNA).^62,63^

### 5.3 Species Difference? Distributions of Protein and Lipid Binding in Plasma

Depending on their structure, PFAS have different affinities to proteins and lipids (Table 2), suggesting that predictive models for plasma binding need to consider the volume fractions of proteins and lipids in plasma. Han et al.^29^ demonstrated that there was no difference between PFOA bound to rat or human serum protein by using ligand blotting. The differences between trout and human plasma of PFBA, PFHxA, PFHpA, PFOA, and PFHxS were obvious in the present study, and the different lipid contents of the two types of plasma are the main cause of the observed species difference, which was also confirmed by the MBMs.

Protein binding of PFAS dominated their binding in human plasma^64^ but not in fish plasma.^65^ PFBA, PFHxA, PFHpA, PFOA, and PFHxS are specifically bound to protein at low concentrations (*ν* < 1), resulting in specific *D*_BSA/w_ or *D*_plasma/w_ almost 10 times higher than their *D*_lip/w_. The volume fraction of lipids in the trout plasma was only half of that of the protein, which was similar to the values reported in a previous study^65^ and decreased the contribution of the specific binding in trout plasma. In contrast to the anionic PFAS, lipid binding was more relevant than protein binding for the neutral FTOHs.

A recent study demonstrated that differences of albumin and globulin contents in human blood affected the free concentrations of PFAS across individuals.^58^ Besides proteins, we also considered the distribution of PFAS to lipids in order to simulate actual plasma conditions. The *D*_plasma/w_ measured in this study can be used in PBTK models to calculate the free PFAS in plasma. For risk assessment, it should also be considered that the amount of proteins and lipids in plasma is influenced by many factors, such as diet, environmental conditions, and health status, which exist not only between species but may also exist between individuals.

## Supporting Information Available

Additional information on chemical structures; derivation of equations; experimental details; binding isotherms and binding constants; and linear relationship of binding constants against the number of perfluorinated carbons (PDF)
