# Supplementary material for: Species Difference? Bovine, Trout, and Human Plasma Protein Binding of Per- and Polyfluoroalkyl Substances
Source: Environ Sci Technol. 2024 May 28;58(23):9954–66. doi: 10.1021/acs.est.3c10824 (PMC11171458; doi:10.1021/acs.est.3c10824)
Supplement: Supplementary file 1 — es3c10824_si_001.pdf [file es3c10824_si_001.pdf]

Species Difference? Bovine, Trout and Human Plasma Protein Binding of Per- and Polyfluoroalkyl Substances

Weiping Qin,[1,2] Beate I. Escher,[1,2] Julia Huchthausen,[1,2] Qiuguo Fu[3] and Luise Henneberger[1]*

[1]Department of Cell Toxicology, UFZ–Helmholtz Centre for Environmental Research, 04318 Leipzig, Germany

[2]Environmental Toxicology, Department of Geosciences, Eberhard Karls University Tübingen, Schnarrenbergstr. 94-96, DE-72076 Tübingen, Germany

[3] Department of Environmental Analytical Chemistry, UFZ–Helmholtz Centre for Environmental Research, 04318 Leipzig, Germany

*Corresponding author: Luise Henneberger − UFZ−Helmholtz Centre for Environmental Research, 04318 Leipzig, Germany; orcid.org/0000-0002-3181-0044; Email: luise.henneberger@ufz.de

Number of pages: 21

Table of content

- **Texts:**

Text S1: Mass balance model for protein/plasma binding of FTOHs. 10

Text S2: Result comparison of PFBA, PFOA, PFHxS and PFOS measured by BioSPME and C18-SPME. 16

- **Tables:**

Table S1 Purchase information and structures of 16 PFAS. 3

Table S2 Concentrations of 13 PFAS in the kinetic uptake experiments, mass balance and experimental conditions for the BioSPME 96-Pin Device. 4

Table S3 LCMS parameters of (a) 12 PFAS and (b, c) HFPO-DA. 5
Table S4 Concentrations of 13 PFAS in sorption isotherm experiment and distribution ratios between pin and water (log $D_{pin/w}$) derived by using Freundlich-type model. 7
Table S5 Concentrations of 16 PFAS in protein and plasma binding assays, as well as bound fractions of PFAS with different mass concentration of BSA (m_{BSA}/V_{total}) or volume concentration of plasma (V_{plasma}/V_{total}) in the assays. 8
Table S6 Experimental parameters of head-space SPME (a) and GCMS (b) for FTOH. 9
Table S7 Partition constants of FTOH between air and water ($K_{air/w}$), glass and air ($K_{glass/air}$). 11
Table S8 Freundlich constant log K_{Fr} and exponent n_{Fr} of 13 PFAS derived from protein and plasmas binding isotherms by using Freundlich-type model (eq 11). 15
Table S9 Binding constants log $D_{i/w}$ (in parentheses 95% confidence intervals) of PFBA, PFOA, PFHxS and PFOS to bovine serum albumin (BSA) and human plasma. 16
Table S10 Protein and plasma concentrations used in the binding assays, as well as bound fractions of PFBA, PFOA, PFHxS and PFOS from two studies. 16

- **Figures:**

Figure S1 Sample preparation of PFAS for binding isotherms to pin, proteins and plasmas 6
Figure S2 Sample preparation of FTOHs for head-space SPME combined with GCMS 9
Figure S3 Linear detector response for the measured GC peak areas (A) and the total concentrations of FTOH (C_{tot}) 11
Figure S4 Sorption kinetic of PFAS to the C18 coating of the pin of the BioSPME 96-Pin Device. 12
Figure S5 Bovine serum albumin (BSA) binding isotherms of 11 PFAS. 14
Figure S6 (a-d) BSA and **(e-h)** human plasma binding isotherms of PFBA, PFOA, PFHxS and PFOS measured by the BioSPME 96-Pin Device and the C18-SPME using single fibers. 17
Figure S7 Human plasma (HP) and trout plasma (TP) binding isotherms of (a) Hexaflumuron and (b) Flubendiamide. 18
Figure S8 Bovine serum albumin (BSA) binding constants of 3 FTOH measured at different concentrations. 18
Figure S9 Plasma binding constants of 16 PFAS plotted against the number (n) of perfluorinated carbons 19
Figure S10 Linear relationship of binding constants of PFAS to liposomes or olive oil (log $D_{lip/w}$ or log $D_{oil/w}$) against the number (n) of perfluorinated carbons 19

- **References**

Table S1 Purchase information and structures of 16 PFAS.

	DTXSID	Name	Abbreviation	Supplier	Structure
**Anionic (10)**					
**1**	DTXSID4059916	Perfluorobutanoic acid	PFBA	J&K Scientific	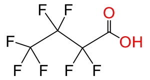
**2**	DTXSID3031862	Perfluorohexanoic acid	PFHxA	Sigma Aldrich	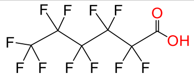
**3**	DTXSID1037303	Perfluoroheptanoic acid	PFHpA	Sigma Aldrich	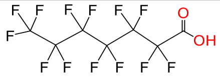
**4**	DTXSID8031865	Perfluorooctanoic acid	PFOA	abcr. GmbH	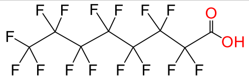
**5**	DTXSID8031863	Perfluorononanoic acid	PFNA	chemPUR	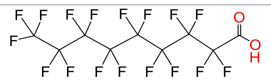
**6**	DTXSID8047553	Perfluoroundecanoic acid	PFUnA	chemPUR	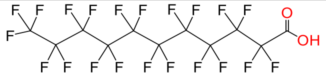
**7**	DTXSID70880215	Perfluoro-2-methyl-3-oxahexanoic acid	HFPO-DA	abcr. GmbH	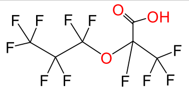
**8**	DTXSID3037709	Perfluorohexane sulfonic acid	PFHxS	Sigma Aldrich	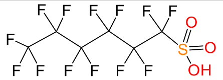
**9**	DTXSID8037706	Perfluorooctane sulfonic acid	PFOS	Sigma Aldrich	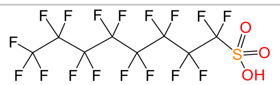
**10**	DTXSID6067331	6:2 Fluorotelomer sulfonic acid	6:2 FTSA	abcr. GmbH	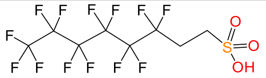
**Partially charged or neutral (3)**					
**11**	DTXSID3038939	Perfluorooctane sulfonamide	PFOSA	HPC Standards GmbH	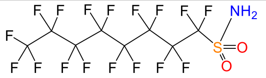
**12**	DTXSID3032620	Hexaflumuron	Hexaflumuron	HPC Standards GmbH	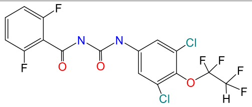
**13**	DTXSID4047672	Flubendiamide	Flubendiamide	HPC Standards GmbH	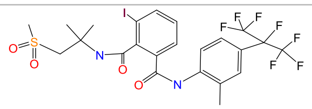
**Neutral and semi-volatile (3)**					
**14**	DTXSID5044572	2-Perfluorohexyl-ethanol	6:2 FTOH	Sigma Aldrich	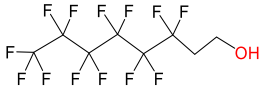
**15**	DTXSID7029904	2-Perfluorooctyl-ethanol	8:2 FTOH	Sigma Aldrich	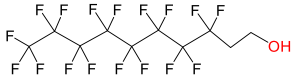
**16**	DTXSID2029905	2-Perfluorodecyl-ethanol	10:2 FTOH	J&K Scientific	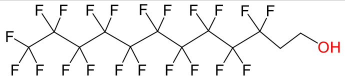

Table S2 Concentrations of 13 PFAS in the kinetic uptake experiments, mass balance and experimental conditions for the BioSPME 96-Pin Device.

	Uptake kinetic assays						Experimental conditions for the BioSPME 96-Pin Device			
**Name**	C_{stock} [mg/mL] (MeOH)	C_{nom} [mg/L] (MeOH)	C_{nom} [mg/L] (Sample)	C_{tot} [mg/L] (Sample-LCMS)	Mass balance (%)	$t_{95\%}$ [min]	Extraction time [min] at 37℃	Desorption time [min] at 25℃	Desorption solvent	Material of the 96-deep well plate used for extraction and desorption
**PFBA**	10	800	8	9.18	103%	<10	30	20	50% Methanol+50% MilliQ water	Plastic
**PFHxA**	10	800	8	9.39	102%	<10	30	20	50% Methanol+50% MilliQ water	Plastic
**PFHpA**	10	800	8	8.78	102%	9	30	20	50% Methanol+50% MilliQ water	Plastic
**PFOA**	10	500	5	5.81	101%	15	30	20	50% Methanol+50% MilliQ water	Plastic
**PFNA**	10	500	5	6.02	103%	26	30	20	100% Methanol	Plastic
**PFUnA**	10	500	5	5.48	115%	60	60	20	100% Methanol	Glass-coated
**HFPO-DA** [a]	/	/	/	/	92%	<30	30	20	50% Methanol+50% MilliQ water	Plastic
**PFHxS**	10	500	5	6.12	98%	15	30	20	50% Methanol+50% MilliQ water	Plastic
**PFOS**	10	500	5	4.59	112%	27	30	20	100% Methanol	Plastic
**6:2 FTSA**	10	500	5	5.27	101%	16	30	20	50% Methanol+50% MilliQ water	Plastic
**PFOSA**	10	500	5	5.27	94%	50	60	20	100% Methanol	Glass-coated
**Hexaflumuron**	5	50	0.5	0.51	93%	15	60	20	100% Methanol	Glass-coated
**Flubendiamide**	5	20	0.1	0.10	102%	57	60	20	100% Methanol	Glass-coated

C_{stock}: Stock solution of PFAS were prepared in methanol.

C_{nom} (MeOH): Nominal concentrations of PFAS stock solutions in methanol.

C_{nom} (Sample): Nominal concentrations of PFAS. All samples contained 1% methanol.

C_{tot} (Sample_LCMS): Total concentrations of PFAS quantified by LCMS.

[a] The mass balance of HFPO-DA was from the fiber sorption isotherms assays and the time to 95% equilibrium $t_{0.95}$ of HFPO-DA less than 30 min was inferred according to the number of carbons in the perfluoroalkane chain, which is the same as PFHxA.

Table S3 LCMS parameters of (a) 12 PFAS and (b, c) HFPO-DA.

**a**		**LC-parameters**				**MS parameters**				**Source**			
**Chemical**	Instrument	Column	Eluent A:B [1]	Injection volume [µL]	Retention time [min]	Ion source	Fragmentor voltage [V]	MRM transition (Collision energy [V])	Calibration range [µg/L]	Gas temperature [°C]	gas flow [L/min]	nebulizer [psi]	capillary voltage [V]
**PFBA**	Agilent Technologies 6420, Triple Quad LC/MS	Phenomenex Luna Omega Polar C18 1.6 µm 50 x 2.1 mm, 25°C	85% : 15%	1	0.70	ESI-	60	212.98 -> 169.0 (4)	5-10000	350	13	45	1500
**PFHxA**			70% : 30%		0.80		86	313->286.8/119 (2/22)	5-10000	270	13	60	2000
**PFHpA**			65% : 35%		0.90		80	362.97 -> 319/169 (4/16)	1-10000	290	12	50	1500
**PFOA**			55% : 45%		0.75		80	412.96 -> 369/169 (8/16)	5-10000	290	13	60	1500
**PFNA**			50% : 50%		0.78		100	462.96 -> 419/219 (4/16)	1-10000	290	12	50	1500
**PFUnA**			40% : 60%		0.88		86	563.0 -> 518.8/268.8 (6/18)	1-10000	310	13	60	2000
**PFHxS**			55% : 45%		0.85		168	398.93 -> 98.9/80 (40/50)	1-10000	350	13	60	1500
**PFOS**			40% : 60%		0.77		135	498.9 -> 98.9/80 (48/50)	1-10000	350	13	60	1500
**6:2 FTSA**			60% : 40%		0.74		152	427 -> 406.8/81 (22/38)	5-10000	350	13	40	3000
**PFOSA**			30% : 70%		0.75		152	497.9 -> 78 (38)	5-10000	350	13	60	1500
**Hexaflumuron**			30% : 70%		0.78		86	461 -> 158/141 (22/40)	0.2-2000	350	13	30	5000
**Flubendiamide**			35% : 65%		0.73		152	681 -> 273.9/253.9 (14/26)	0.2-2000	350	13	60	5000

[a] Eluent A: 95% MilliQ water + 5% acetonitrile + 0.1% formic acid; Eluent B: 5% MilliQ water + 95% acetonitrile + 0.1% formic acid;

**b**		**LC-parameters**				**MS parameters**			**Source**			
**Chemical**	Instrument	Column	Eluent A:B	Injection volume [µL]	Retention time [min]	Ion source	m/z	Calibration range [µg/L]	Transfer capillary [°C]	Sheath gas flow rate	Auxiliary gas flow rate	Spray voltage [V]
**HFPO-DA**	Thermo Ultimate 3000 LC system, an electrospray ion source and a Q Exactive Plus quadrupole-orbitrap instrument	ACQUITY UPLC® BEH C18, 100 x 2 mm, 1.7 µm particle size	Solvent gradients (bellow)	5	12.92	ESI-	328.9677	0.1-1000	300	45 a.u.	1 a.u.	3500

**c**	**Eluent A: final % (2 mM ammonium bicarbonate in water)**	**Eluent B: final % (2 mM ammonium bicarbonate in 95:5 MeOH:water)**
**0 min**	100	0
**15 min**	0	100
**22 min**	100	0
**30 min**	100	0

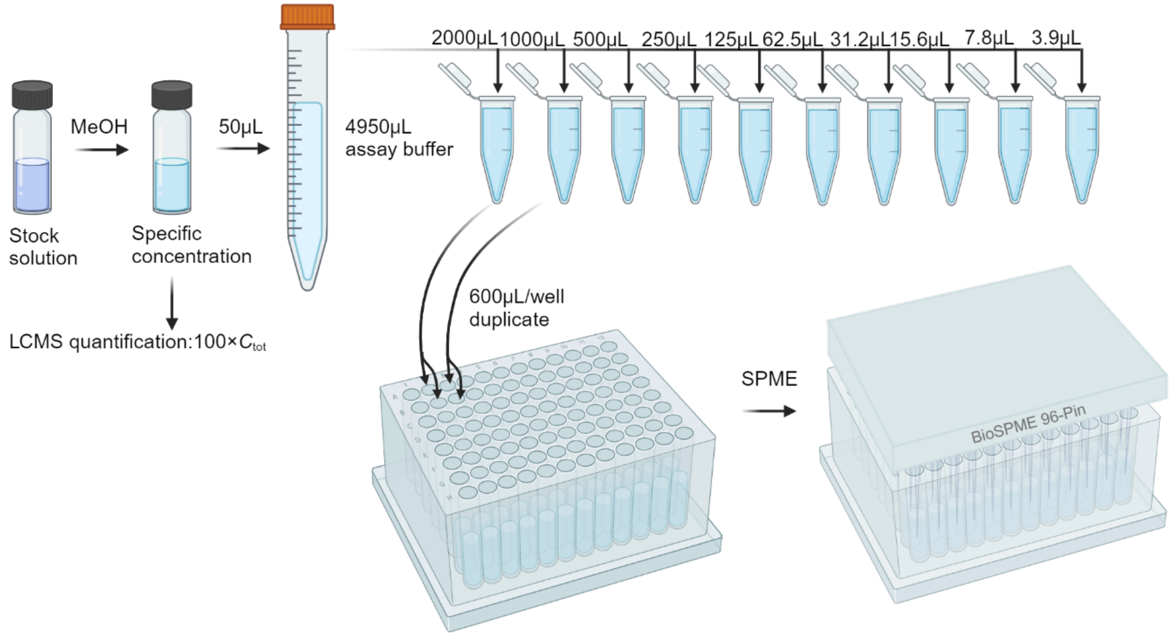

Figure S1 Sample preparation of PFAS for binding isotherms to pin, proteins and plasmas

Table S4 Concentrations of 13 PFAS in sorption isotherm experiment and distribution ratios between pin and water (log $D_{pin/w}$) derived by using Freundlich-type model.

The Freundlich constant log K_{Fr} and exponent n_{Fr} were derived by using a Freundlich-type model (eq 10). For PFAS with $n_{Fr} > 0.9$, log $D_{pin/w}$ were equal to log K_{Fr} by setting $n_{Fr} = 1$. Regression equations of log $D_{pin/w}$ against the concentration of PFAS in C18-partical coating of the pin (log C_{pin} [µmol/L_{pin}] with V_{pin} = 80 nL) were derived by eq 10 if the sorption isotherm is concentration-dependent.

**Name**	**C_{tot} [µmol/L] (Sample_LC MS)**	**Freundlich-type Model**			**log $D_{pin/w}$[L_w/L_{pin}]**	**R^2**
		n_{Fr}	log K_{Fr}	log K_{Fr} (n_{Fr}= 1)		
PFBA	997.8	0.73	1.47		log $D_{pin/w}$=-0.378×log C_{pin}+2.02	0.71
PFHxA	102.4	0.84	2.17		log $D_{pin/w}$=-0.189×log C_{pin}+2.58	0.81
PFHpA[a]	17.0	0.93	2.71		log $D_{pin/w}$=-0.079×log C_{pin}+2.92	0.48
PFOA	10.7	0.85	3.46		log $D_{pin/w}$=-0.177×log C_{pin}+4.07	0.59
PFNA	14.8	0.96	3.77	3.79	3.79	0.98
PFUnA	12.9	1.01	4.17	4.16	4.16	0.99
HFPO-DA	182.9	0.69	2.71		log $D_{pin/w}$=-0.439×log C_{pin}+3.91	0.61
PFHxS	7.5	0.82	3.05		log $D_{pin/w}$=-0.227×log C_{pin}+3.74	0.62
PFOS	10.6	0.81	3.75		log $D_{pin/w}$=-0.240×log C_{pin}+4.65	0.45
6:2 FTSA	15.0	0.86	3.42		log $D_{pin/w}$=-0.164×log C_{pin}+3.98	0.56
PFOSA	14.5	0.91	3.88	3.92	3.92	0.98
Hexaflumuron	2.6	1.05	4.17	4.08	4.08	0.96
Flubendiamide	2.0	0.90	4.17	4.36	4.36	0.95

[a] PFHpA: log C_{pin} of PFHpA was presented in a concentration-dependent way although its n_{Fr} = 0.93

Table S5 Concentrations of 16 PFAS in protein and plasma binding assays, as well as bound fractions of PFAS with different mass concentration of BSA (m_{BSA}/V_{total}) or volume concentration of plasma (V_{plasma}/V_{total}) in the assays.

		**Protein binding assays**		**Plasma binding assays**		
**Name**	C_{tot} [µmol/L] (Sample LCMS)	Mass concentration of BSA [mg/L] in assay	BSA f_{bound}	Volume concentration of plasma [mL/L] in assay	Human plasma f_{bound}	Fish plasma f_{bound}
**PFBA** [a]	2712	5000	46%	100	35%	2%
**PFHxA** [a]	731	1500	59%	30	58%	17%
**PFHpA** [a]	78	500	65%	10	65%	34%
**PFOA** [a]	100	500	87%	10	77%	72%
**PFNA** [a]	100	500	86%	10	79%	86%
**PFUnA** [a]	76	500	87%	10	84%	89%
**HFPO-DA** [a]	203	1500	58%	30	42%	52%
**PFHxS** [a]	54	100	57%	2	53%	31%
**PFOS** [a]	55	100	72%	2	64%	71%
**6:2 FTSA** [a]	106	500	65%	10	62%	67%
**PFOSA** [a]	96	500	78%	10	63%	64%
**Hexaflumuron** [a]	29	500	69%	10	77%	86%
**Flubendiamide** [a]	37	500	44%	10	51%	65%
**6:2 FTOH** [b]	4.5	10000	42%	100	35%	22%
**8:2 FTOH** [b]	0.45	10000	62%	100	49%	58%
**10:2 FTOH** [b]	0.045	10000	79%	100	51%	70%

[a] The bound fraction of 13 PFAS were the average values of all concentration points in the binding isotherms. Samples were measured by BioSPME combined with LCMS.

[b] The bound fraction of 3 FTOH were the average values of all samples, which were measured individually by head-space SPME combined with GCMS.

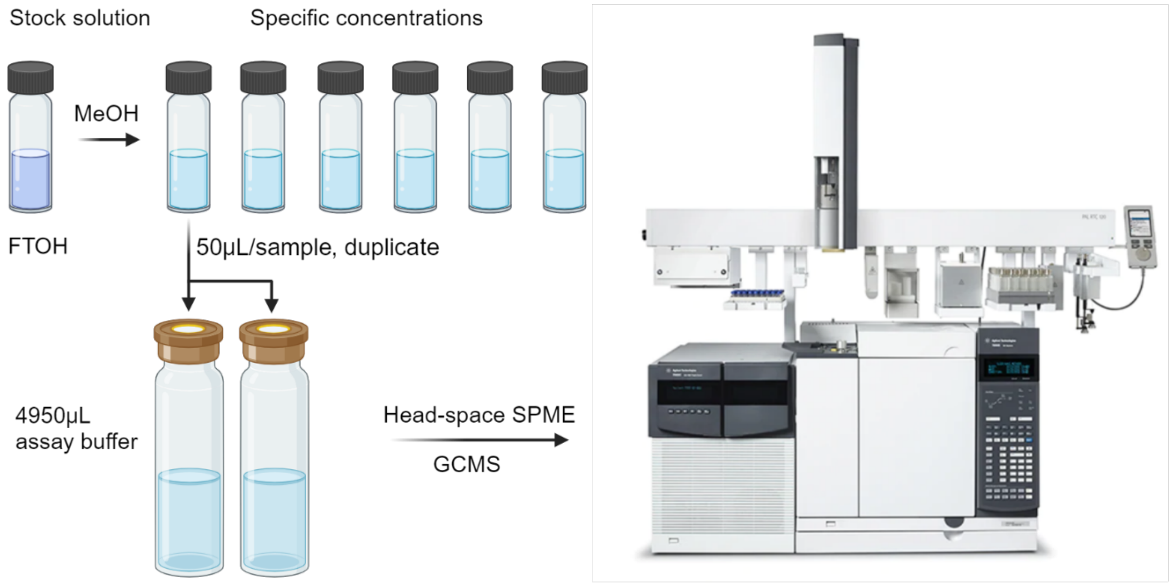

Figure S2 Sample preparation of FTOHs for head-space SPME combined with GCMS.

Table S6 Experimental parameters of head-space SPME (a) and GCMS (b) for FTOH.

**a**	**Autosampler**	**Incubation**	**Extraction**	**Vial penetration**	**Injector penetration**	**Injection**
**HS-SPME**	MPS robotic XL (Gerstel) installed to the GC-MS system and controlled by Maestro Control software V1.4	10 min, 37°C, 250rpm	30 min, 37°C, 250rpm	21 mm	45 mm	3min, 230°C, splitless

**b**	**GCMS**	**GC edit parameter**			**MS**		
		Split-splitless inlet	column	oven	SIM (m/z)	Retention time	Tune type: CI
**6:2 FTOH**	Agilent 8890 GC coupled to a 5977B GC/MSD (Agilent, Waldbronn, Germany)	heater 220°C, septum purge flow 3mL/min, purge flow to split vent 50ml/min at 3.2 min	CP-WAX 57 CB column (25m x 0.25 mm x 0.2 μm, Agilent) with carrier gas at a constant flow of 1.1 mL/min	Gradient temperature: 50-150 °C, rate 20°C/min, hold time: 3.2min (50°C) and 2min(150°C), rum time 10.2 min	365.1	6.1	CI Gas Valve: A; CI Flow: 20%; solvent delay 3min; EM
**8:2 FTOH**					465.2	6.9	
**10:2 FTOH**					565.1	7.6	

Text S1: Mass balance model for protein/plasma binding of FTOHs.

In a closed head-space vial, the total amount of FTOH (n_{tot}) partitions between water (n_w), air (n_{air}), wet-glass surface (n_{glass}) and biomaterials ($n_{bound,i}$, i=BSA, plasma proteins and lipids).

$$n_{tot}=n_w+n_a+n_{glass}+n_{bound,i} \tag{S1}$$

The amount of FTOH in different phases can be related to the free concentration of FTOH in aqueous phase (C_w) with the partition constants of FTOH between air and water ($K_{air/w}$), wet-glass surface and air ($K_{glass/air}$), biomaterials and water ($D_{i/w}$, i=BSA, plasma) as eq S2.

$$n_w=C_w\times V_w$$
$$n_a=C_{air}\times V_{air}=C_w\times K_{air/w}\times V_a$$
$$n_{glass}=C_{glass}\times S_{glass}=C_w\times K_{air/w}\times K_{glass/air}\times S_{glass}$$
$$n_{bound,i}=C_{bound,i}\times V_i=C_w\times D_{i/w}\times V_i \tag{S2}$$

Inserting eq S2 to eq S1, yields eq S3 for C_w of FTOH in aqueous phase of PBS samples as control, as well as eq S4 for $C_{w,i}$ of FTOH in aqueous phase of BSA and plasma samples. n_{tot} of FTOH used in control, BSA, plasma samples were same in parallel.

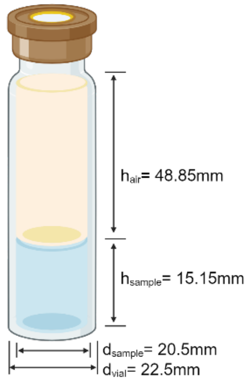

$$C_w=\frac{n_{tot}}{V_w+K_{air/w}\times V_{air}+K_{air/w}\times K_{glass/air}\times S_{glass}} \tag{S3}$$

$$C_{w,i}=\frac{n_{tot}}{V_w+K_{air/w}\times V_{air}+K_{air/w}\times K_{glass/air}\times S_{glass}+D_{i/w}\times V_i} \tag{S4}$$

The area of wet-glass surface is calculated for 20 mL head-space vial according to the vial parameters that measured in this assay.

$$S=\pi d_{sample}\times h_{air}+\pi\left(\frac{d_{sample}}{2}\right)^2\times 2=3.806\times 10^{-3}\ (m^2) \tag{S5}$$

The log $K_{air/w}$ of FTOHs at 25°C were from Goss, et al.[1] and the wet-glass bound fraction was already considered for the derivation of the log $K_{air/w}$. Since all samples were conditioned in autosampler and extracted by head-space SPME at 37°C, the values of $K_{air/w}$ need to be further extrapolated to the values at 37°C by eq S6.

$$K_{i12}^{T2} = K_{i12}^{T1}\times \exp\left[-\frac{\Delta_{12}H_i}{R}\times\left(\frac{1}{T_2}-\frac{1}{T_1}\right)\right] \tag{S6}$$

The temperatures were T_1=298K (25°C) and T_2=310K (37°C). The enthalpy (Δ H) of FTOH were from LSER database.[2] Extrapolation of $K_{air/w}$ at 37°C were listed in Table S7. The log $K_{glass/air}$ of FTOH at 35°C were from Arp et al.[3] The temperature was not much different from our experimental condition and thus the values were directly used. The log $K_{glass/air}$ was used to include the bound fraction of wet-glass surface in the head-space vials for mass balance in eq S10.

Table S7 Partition constants of FTOH between air and water ($K_{air/w}$), glass and air ($K_{glass/air}$).

	$K_{air/w}$ at 25°C [1]	Δ H (J/mol) [2]	$K_{air/w}$ at 37°C	log $K_{glass/air}$ at 35°C [3]
6:2 FTOH	-0.56	7.90E+04	-0.02	-2.62
8:2 FTOH	0.58	8.95E+04	1.19	-1.92
10:2 FTOH	1.60	9.98E+04	2.28	-1.30

The measured GC peak areas (A) are linearly related to the total concentrations (C_{tot}) of FTOH and the regression equations are depicted in Figure S3. Given a linear detector response, the GC peak areas of the FTOHs from the control samples (A_w, eq S7), and BSA or plasma samples ($A_{w,i}$, eq S8) can be assumed to be linearly related to the concentration of the FTOH in the aqueous phases.

$$A_w = slope \times C_w \tag{S7}$$

$$A_{w,\ i} = slope \times C_{w,i} \tag{S8}$$

A ratio of GC peak areas of FTOH can relate to the aqueous concentration in BSA or plasma samples and control samples as eq S9 by cancelling the slope of GC response factor. Insertion of eqs S3 and S4 into eq S9, yields eq S10 after cancelling the n_{tot} and moving the $D_{i/w}$ to left side of the equation.

$$\frac{A_w}{A_{w,\ i}} = \frac{C_w}{C_{w,i}} = \frac{V_w + K_{air/w} \times V_{air} + K_{air/w} \times K_{glass/air} \times S_{glass} + D_{i/w} \times V_i}{V_w + K_{air/w} \times V_{air} + K_{air/w} \times K_{glass/air} \times S_{glass}} \tag{S9}$$

$$D_{i/w} = \frac{1}{V_i} \times [(\frac{A_w}{A_{w,\ i}} - 1) \times \left(V_w + K_{a/w} \times V_a + K_{a/w} \times K_{glass/a} \times S_{glass}\right)] \tag{S10}$$

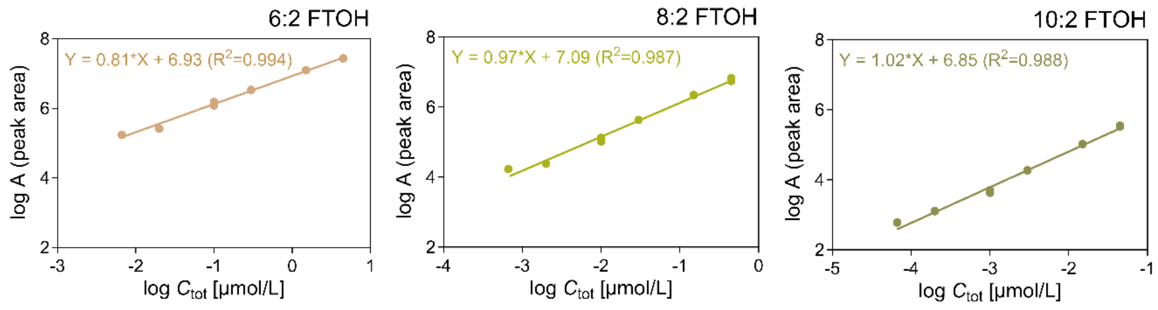

Figure S3 Linear detector response for the measured GC peak areas (A) and the total concentrations of FTOH (C_{tot})

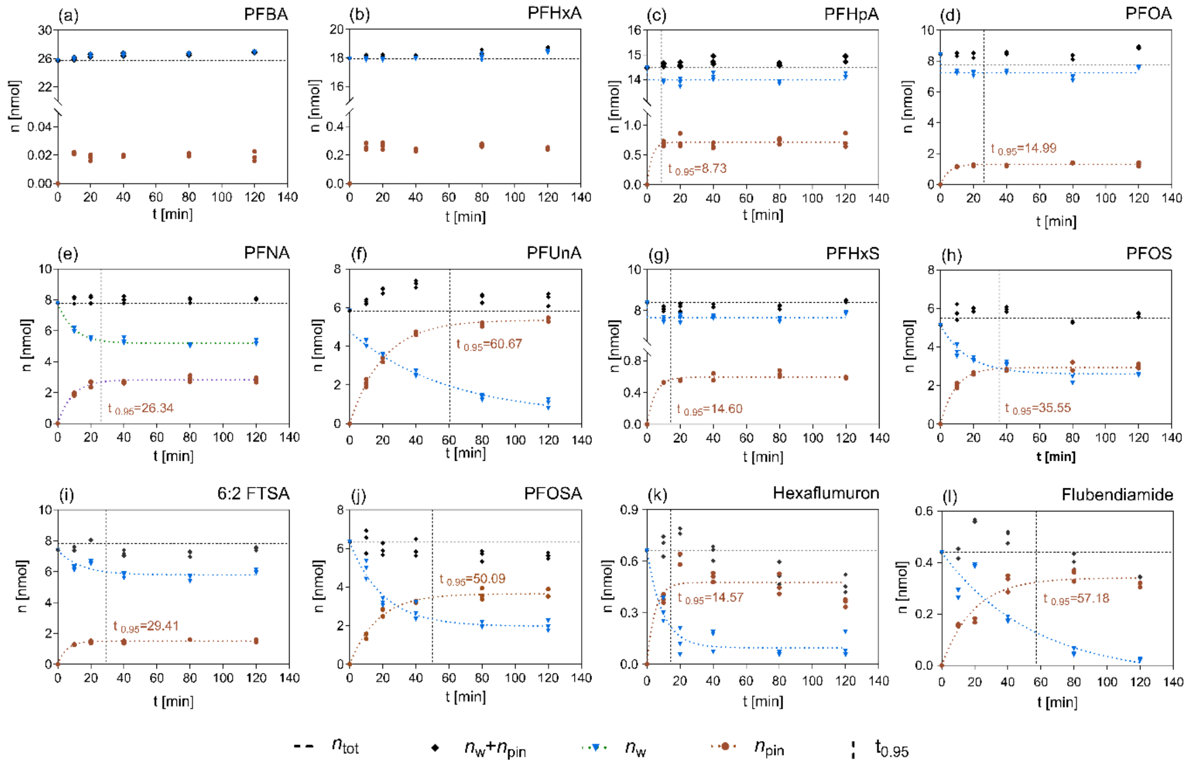

Figure S4 Sorption kinetic of PFAS to the C18 coating of the pin of the BioSPME 96-Pin Device.

The black dashed horizontal line is the total amount of chemical (n_{tot}) used in the samples. n_w (blue triangles) and n_{pin} (red dots) were the measured molar amount of chemicals in the water phase or pin coating. The sum of n_w (eq 1) and n_{pin} (eq 2) (black diamonds) were compared with n_{tot} to derive the mass balance (eq 3). $t_{0.95}$ (eq 6) is the time to reach 95% equilibrium and is indicated by a black dashed vertical line.

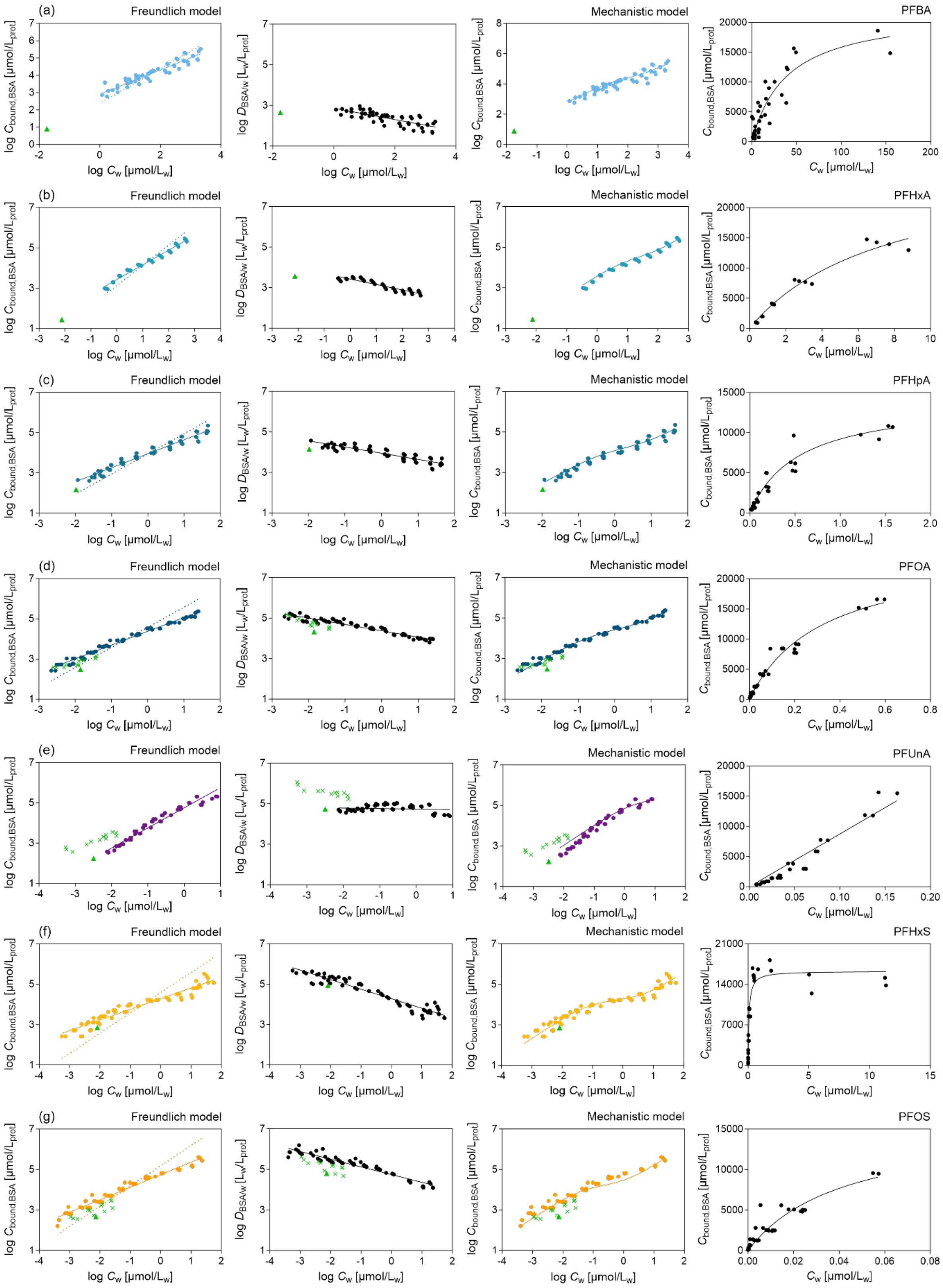

Figure S5 Continued bovine serum albumin (BSA) binding isotherms of 11 PFAS.

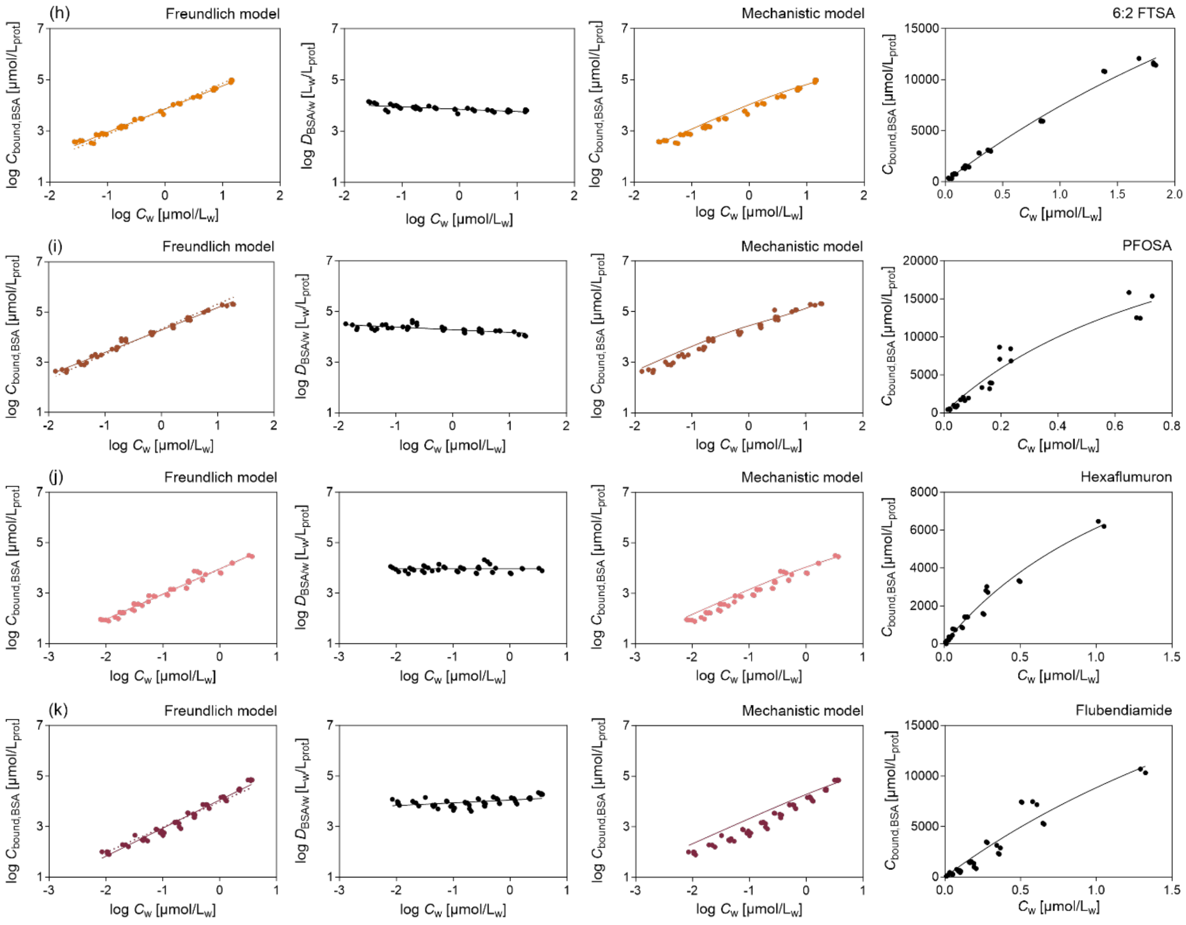

Figure S5 Bovine serum albumin (BSA) binding isotherms of 11 PFAS.

Figures in first column: BSA binding isotherms were fitted linearly with Freundlich model (eq 8, solid line), the dotted line was developed by setting $n_{Fr} = 1$. Figures in the second column: the concentration-dependent distribution ratios between BSA and water, log $D_{BSA/w}$ were fitted log-log linear (eq 11). Figures in the third column: data points were fitted non-linearly with combined binding/partition model (eq 15), Figures in the fourth column: the saturable specific-binding in low concentration rages was derived with eq 13. Results in this study were compared with the results from literature[4, 5] (green triangles and crosses).

Table S8 Freundlich constant log K_{Fr} and exponent n_{Fr} of 13 PFAS derived from protein and plasmas binding isotherms by using Freundlich-type model (eq 11).

log $D_{i/w}$ (i = Bovine serum albumin-BSA, human plasma and trout plasma) were equal to log K_{Fr} by setting $n_{Fr} = 1$ if the binding isotherm is weakly dependent on concentrations ($0.90 < n_{Fr} < 1$) or independent of concentrations ($n_{Fr} \geq 1$).

	**BSA: log $D_{BSA/w}$ [L_w/L_{prot}]**			**Human plasma: log $D_{plasma/w}$ [$L_w/L_{prot+lip}$]**			**Trout plasma: log $D_{plasma/w}$ [$L_w/L_{prot+lip}$]**		
	n_{Fr}	log K_{Fr}	log K_{Fr} ($n_{Fr} = 1$)	n_{Fr}	log K_{Fr}	log K_{Fr} (n_{Fr}=1)	n_{Fr}	log K_{Fr}	log K_{Fr} ($n_{Fr} = 1$)
**PFBA**	0.70	2.91		0.60	2.80		**0.71**[a]	1.95	1.43
**PFHxA**	0.73	3.43		0.71	3.29		**0.94**	2.55	2.49
**PFHpA**	0.70	3.95		0.61	3.79		**1.19**	3.23	3.32
**PFOA**	0.69	4.38		0.59	4.02		**0.89** [a]	4.16	4.18
**PFNA**	0.85	4.52		0.82	4.13		**0.90**	4.78	4.71
**PFUnA**	**0.97**	4.73	4.75	**0.90**	4.47	4.54	**0.93**	4.94	4.99
**HFPO-DA**	0.51	3.44		0.37	3.41		0.35	3.20	
**PFHxS (K)**	0.53	4.28		0.32	3.95		0.76	3.96	
**PFOS (K)**	0.62	4.74		0.66	4.46		0.70	4.91	
**6:2 FTSA**	0.90	3.86		0.86	3.66		**0.88** [a]	4.10	4.11
**PFOSA**	0.89	4.28		0.89	3.87		0.84	4.26	
**Hexaflumuron**	**1.00**	3.96	3.96	**1.23**	4.52	4.29	**1.00**	4.61	4.61
**Flubendiamide**	**1.11**	4.05	3.98	**1.06**	3.93	3.88	**1.10**	4.56	4.46

[a] Log $D_{plasma/w}$ of PFBA, PFOA, 6:2 FTSA were set to be log K_{Fr} even though their $n_{Fr} < 0.90$, because their binding isotherms of trout plasma were observed independent of concentrations in Figure 2.

Text S2: Result comparison of PFBA, PFOA, PFHxS and PFOS measured by BioSPME and C18-SPME**.**

Protein and plasma binding isotherms of PFBA, PFOA, PFHxS and PFOS were measured in this study by the BioSPME 96-Pin Device and competed to our previous study where they were measured by C18-SPME using single fibers.[6] Here, we provide more information about the comparison of results (Table S9) and experimental conditions (Table S10) of the two assay formats.

Table S9 Binding constants log $D_{i/w}$ (in parentheses 95% confidence intervals) of PFBA, PFOA, PFHxS and PFOS to bovine serum albumin (BSA) and human plasma.

	**Protein binding: log $D_{BSA/w}$ [L_w/L_{prot}]**		**Plasma binding: log $D_{plasma/w}$ [$L_w/L_{prot+lip}$]**		**Source (assay method)**
	Specific	Non-specific	Specific	Non-specific	
**PFBA**	2.44 (2.10-2.63)	1.97 (1.90-2.04)	2.20 (0.87-2.49)	1.35 (1.23-1.44)	This study (BioSPME)
**PFOA**	4.58 (4.47-4.67)	3.88 (3.86-3.90)	4.45 (4.34-4.54)	3.65 (3.59-3.70)	
**PFHxS**	5.02 (4.90-5.11)	3.58 (3.50-3.64)	4.98 (4.80-5.11)	2.92 (2.78-3.03)	
**PFOS**	5.27 (4.84-5.48)	4.17 (4.14-4.20)	4.82 (4.41-5.03)	4.07 (4.04-4.11)	
**PFBA**	2.40 (/ -2.93)	1.63 (1.56-1.68)	2.12 (1.02-2.44)	1.34 (0.80-1.58)	Qin, et al. 2023 (C18-SPME)
**PFOA**	4.60 (4.27-4.79)	3.70 (3.69-3.72)	4.60 (4.43-4.72)	3.21 (3.13-3.28)	
**PFHxS**	5.10 (4.94-5.22)	3.47 (3.45-3.49)	5.18 (5.05-5.27)	2.82 (2.74-2.89)	
**PFOS**	5.81 (5.11-6.15)	4.84 (4.75-4.92)	5.96 (5.26-6.22)	4.69 (4.61-4.76)	

Table S10 Protein and plasma concentrations used in the binding assays, as well as bound fractions of PFBA, PFOA, PFHxS and PFOS from two studies.

	**Mass concentration of BSA [mg/L]**	**Bound fraction**	**Volume concentration of plasma [mL/L]**	**Bound fraction**	**Source (assay method)**
**PFBA**	5000	25%-63%	100	11%-66%	This study (BioSPME)
**PFOA**	500	70%-95%	10	55%-93%	
**PFHxS**	100	25%-90%	2	20%-93%	
**PFOS**	100	40%-87%	2	39%-82%	
**PFBA**	5000	15%-70%	100	15%-51%	Qin, et al. 2023 (C18-SPME)
**PFOA**		93%-99.7%		71%-95%	
**PFHxS**		90%-99.7%		75%-99%	
**PFOS**		98.4-99.9%		90%-99.9%	

As shown in Figure S6, the binding isotherms of PFBA, PFOA, PFHxS from two methods were very consistent. Thus, all data points of PFBA, PFOA and PFHxS from the two methods were fitted together to derive $D_{BSA/w}$ and $D_{plasma/w}$ (Table 2). It is possible to use the BioSPME 96-Pin Device to measure 96 samples in a high-throughput way. However, the difference of PFOS in the specific binding of protein and plasma were obvious, which may be caused by the different protein concentrations used in the assays. A comparison of experimental conditions of the experiments with four PFAS is given in Table S10. 5000 mg/L BSA were used previously, resulting a binding fraction of PFOS more than 99% at low concentrations. The binding fraction of PFOS was between 40-90% after adjusting BSA to 100 mg/L in the present study.

As for derivation the protein binding constants of PFOS, the last four data points in the low concentration range were excluded for the full fit of the binding isotherms (Figure S6d). Similar situation was found in the plasma binding of PFOS. 100 mL/L human plasma had extreme high bound fractions for several points in low concentration range, which were excluded when fitting the plasma binding isotherms (Figure S6h).

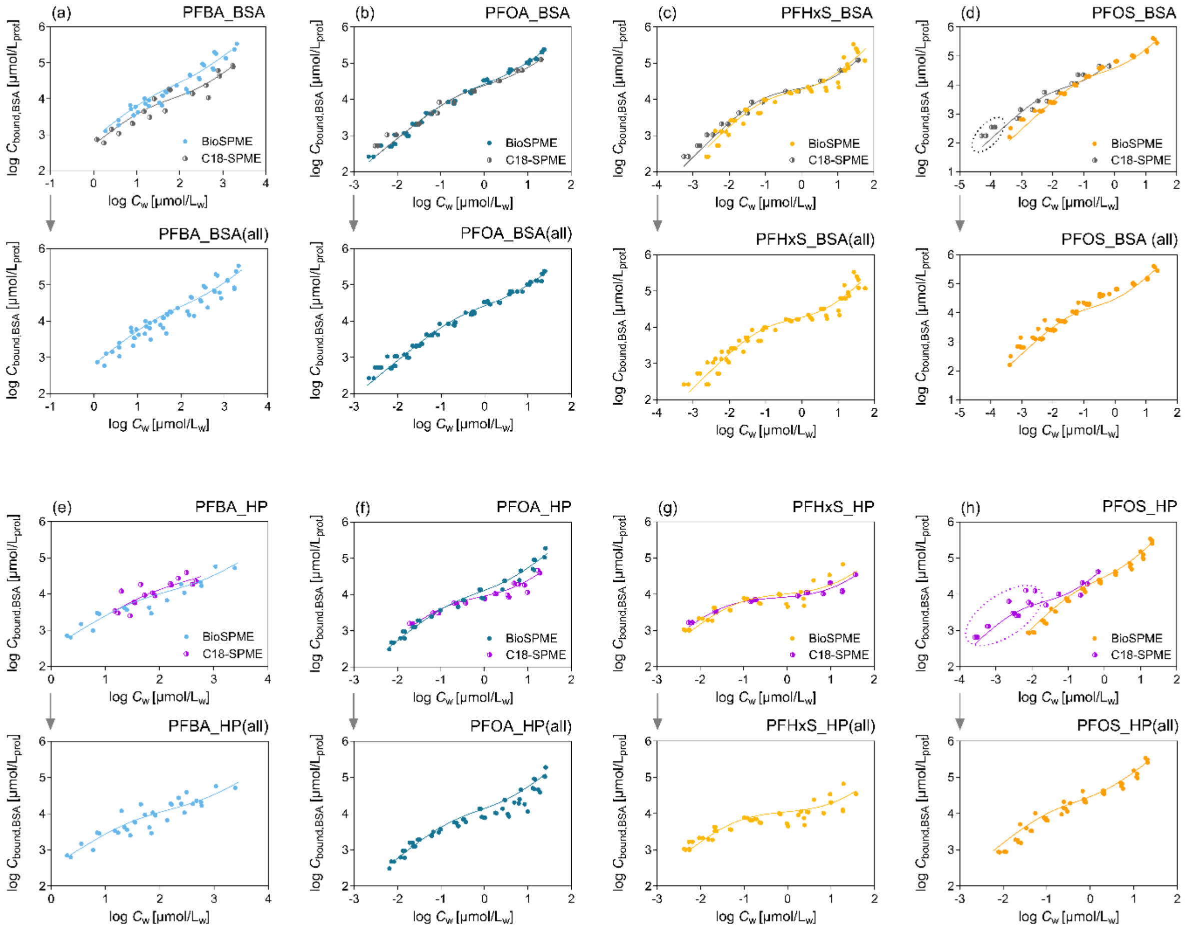

Figure S6 (a-d) BSA and **(e-h)** human plasma binding isotherms of PFBA, PFOA, PFHxS and PFOS measured by the BioSPME 96-Pin Device and the C18-SPME using single fibers.

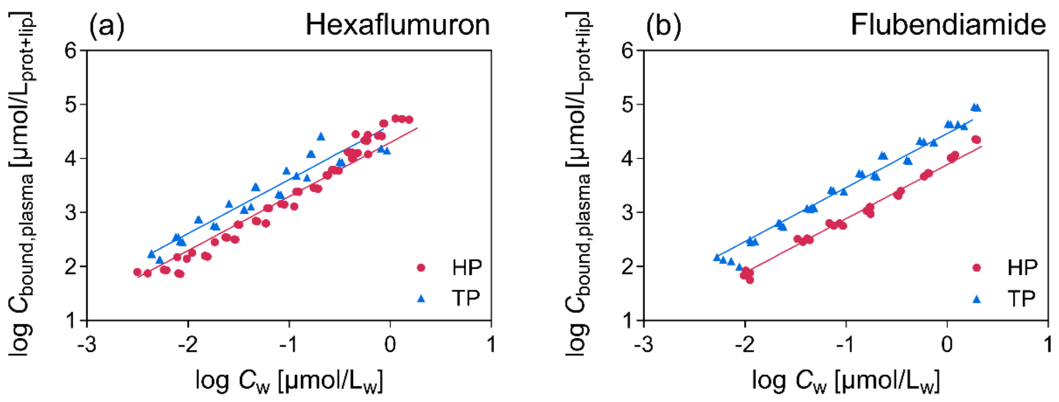

Figure S7 Human plasma (HP) and trout plasma (TP) binding isotherms of (a) Hexaflumuron and (b) Flubendiamide.

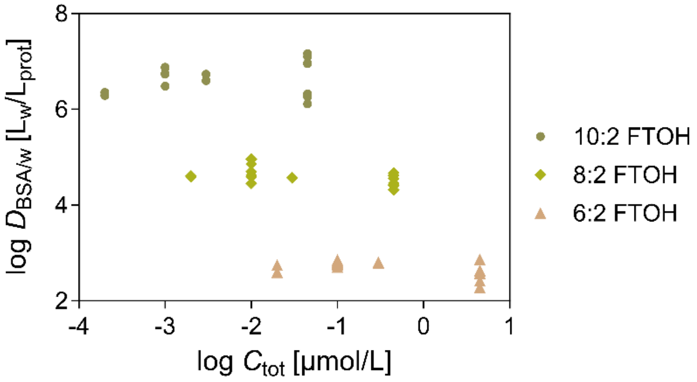

Figure S8 Bovine serum albumin (BSA) binding constants of 3 FTOH measured at different concentrations.

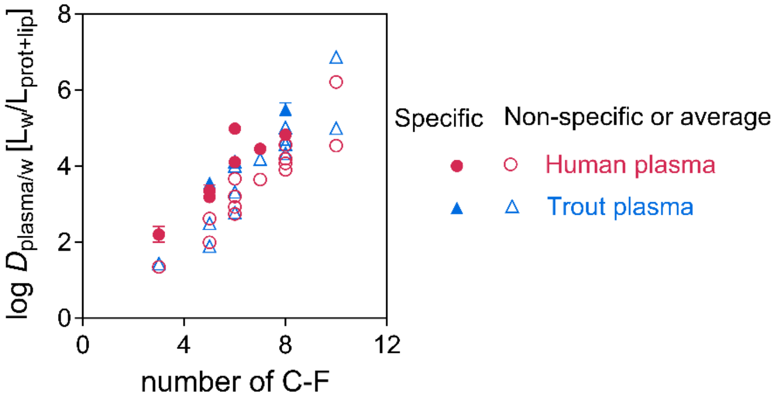

Figure S9 Plasma binding constants of 16 PFAS plotted against the number (n) of perfluorinated carbons

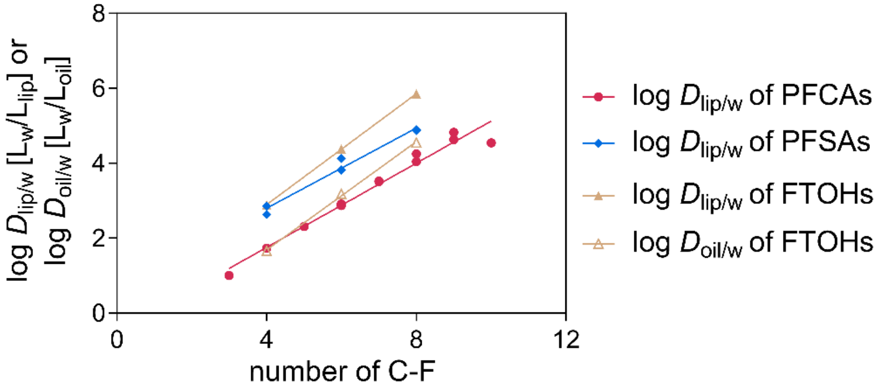

Figure S10 Linear relationship of binding constants of PFAS to liposomes or olive oil (log $D_{lip/w}$ or log $D_{oil/w}$) against the number (n) of perfluorinated carbons

Log $D_{lip/w}$ of perfluoroalkyl carboxylic acid (PFCA), perfluoroalkyl sulfonic acid (PFSA) and fluorotelomer alcohol were measured by Droge et al.,[7] Ebert et al.[8] and Endo et al.[9] Linear regressions of log $D_{lip/w}$ against the number (n) of perfluorinated carbons were fitted for PFCA (magenta circles, eq S11), PFSA (blue diamonds, eq S12) and FTOH (gold filled triangles, eq S13). Log $D_{oil/w}$ of FTOH were measured by Endo et al.[9] Linear regressions of log $D_{oil/w}$ against the number (n) of perfluorinated carbons were fitted for FTOH (gold empty triangles, eq S14).

PFCA: $\log D_{lip/w}=0.562\times n-0.494$ ($R^2=0.969$) (S11)

PFSA: $\log D_{lip/w}=0.535\times n+0.658$ ($R^2=0.977$) (S12)

FTOH: $\log D_{lip/w}=0.740\times n-0.067$ ($R^2=0.999$) (S13)

FTOH: $\log D_{oil/w}=0.723\times n-1.21$ ($R^2=0.999$) (S14)

References

(1) Goss, K. U.; Bronner, G.; Harner, T.; Hertel, M.; Schmidt, T. C. The partition behavior of fluorotelomer alcohols and olefins. *Environ Sci Technol* **2006**, *40* (11), 3572-3577. DOI: 10.1021/es060004p.

(2) Ulrich, N.; Endo, S.; Brown, T. N.; Watanabe, N.; Bronner, G.; Abraham, M. H.; Goss, K.-U. UFZ-LSER database v 3.2.1 [Internet], Leipzig, Germany, Helmholtz Centre for Environmental Research-UFZ. **2017**. Available from http://www.ufz.de/lserd, [accessed on 17.12.2023].

(3) Arp, H. P.; Niederer, C.; Goss, K. U. Predicting the partitioning behavior of various highly fluorinated compounds. *Environ Sci Technol* **2006** *40* (23), 7298-7304. DOI: 10.1021/es060744y.

(4) Allendorf, F.; Berger, U.; Goss, K. U.; Ulrich, N. Partition coefficients of four perfluoroalkyl acid alternatives between bovine serum albumin (BSA) and water in comparison to ten classical perfluoroalkyl acids. *Environ Sci Process Impacts* **2019**, *21* (11), 1852-1863. DOI: 10.1039/c9em00290a.

(5) Xia, X.; Rabearisoa, A. H.; Jiang, X.; Dai, Z. Bioaccumulation of perfluoroalkyl substances by Daphnia magna in water with different types and concentrations of protein. *Environ Sci Technol* **2013**, *47* (19), 10955-10963. DOI: 10.1021/es401442y.

(6) Qin, W.; Henneberger, L.; Huchthausen, J.; Konig, M.; Escher, B. I. Role of bioavailability and protein binding of four anionic perfluoroalkyl substances in cell-based bioassays for quantitative in vitro to in vivo extrapolations. *Environ Int* **2023**, *173*, 107857. DOI: 10.1016/j.envint.2023.107857.

(7) Droge, S. T. J. Membrane-Water Partition Coefficients to Aid Risk Assessment of Perfluoroalkyl Anions and Alkyl Sulfates. *Environ Sci Technol* **2019**, *53* (2), 760-770. DOI: 10.1021/acs.est.8b05052.

(8) Ebert, A.; Allendorf, F.; Berger, U.; Goss, K. U.; Ulrich, N. Membrane/Water Partitioning and Permeabilities of Perfluoroalkyl Acids and Four of their Alternatives and the Effects on Toxicokinetic Behavior. *Environ Sci Technol* **2020**, *54* (8), 5051-5061. DOI: 10.1021/acs.est.0c00175.

(9) Endo, S.; Goss, K. U. Predicting partition coefficients of Polyfluorinated and organosilicon compounds using polyparameter linear free energy relationships (PP-LFERs). *Environ Sci Technol* **2014**, *48* (5), 2776-2784. DOI: 10.1021/es405091h.
